# Segmented flow generator for serial crystallography at the European X-ray free electron laser

**DOI:** 10.1038/s41467-020-18156-7

**Published:** 2020-09-09

**Authors:** Austin Echelmeier, Jorvani Cruz Villarreal, Marc Messerschmidt, Daihyun Kim, Jesse D. Coe, Darren Thifault, Sabine Botha, Ana Egatz-Gomez, Sahir Gandhi, Gerrit Brehm, Chelsie E. Conrad, Debra T. Hansen, Caleb Madsen, Saša Bajt, J. Domingo Meza-Aguilar, Dominik Oberthür, Max O. Wiedorn, Holger Fleckenstein, Derek Mendez, Juraj Knoška, Jose M. Martin-Garcia, Hao Hu, Stella Lisova, Aschkan Allahgholi, Yaroslav Gevorkov, Kartik Ayyer, Steve Aplin, Helen Mary Ginn, Heinz Graafsma, Andrew J. Morgan, Dominic Greiffenberg, Alexander Klujev, Torsten Laurus, Jennifer Poehlsen, Ulrich Trunk, Davide Mezza, Bernd Schmidt, Manuela Kuhn, Raimund Fromme, Jolanta Sztuk-Dambietz, Natascha Raab, Steffen Hauf, Alessandro Silenzi, Thomas Michelat, Chen Xu, Cyril Danilevski, Andrea Parenti, Leonce Mekinda, Britta Weinhausen, Grant Mills, Patrik Vagovic, Yoonhee Kim, Henry Kirkwood, Richard Bean, Johan Bielecki, Stephan Stern, Klaus Giewekemeyer, Adam R. Round, Joachim Schulz, Katerina Dörner, Thomas D. Grant, Valerio Mariani, Anton Barty, Adrian P. Mancuso, Uwe Weierstall, John C. H. Spence, Henry N. Chapman, Nadia Zatsepin, Petra Fromme, Richard A. Kirian, Alexandra Ros

**Affiliations:** 1 School of Molecular Sciences, Arizona State University, Tempe, AZ 85287-1604 USA; 2 Center for Applied Structural Discovery, The Biodesign Institute, Arizona State University, Tempe, AZ 85287-7401 USA; 3 European XFEL, Holzkoppel 4, 22869 Schenefeld, Germany; 4 Department of Physics, Arizona State University, Tempe, AZ 85287-1504 USA; 5 Institute for X-Ray Physics, University of Göttingen, Friedrich-Hund-Platz 1, 37077 Göttingen, Germany; 6 Deutsches Elektronen-Synchrotron, Notkestrasse 85, 22607 Hamburg, Germany; 7 Centre for Ultrafast Imaging, Universität Hamburg, Luruper Chaussee 149, 22761 Hamburg, Germany; 8 Center for Free-Electron Laser Science, Deutsches Elektronen-Synchrotron, Notkestrasse 85, 22607 Hamburg, Germany; 9 Department of Physics, Universität Hamburg, Luruper Chaussee 149, 22761 Hamburg, Germany; 10 Hamburg University of Technology, Vision Systems E-2, Harburger Schloßstraße 20, 21079 Hamburg, Germany; 11 Division of Structural Biology, University of Oxford, Oxford, OX1 2JD United Kingdom; 12 Diamond Light Source Ltd, Didcot, Oxfordshire OX11 0DE United Kingdom; 13 Paul Scherrer Institute, Forschungsstrasse 111, 5232 Villigen, Switzerland; 14 School of Chemical and Physical Sciences, Keele University, Staffordshire, ST5 5AZ United Kingdom; 15 Department of Structural Biology, Jacobs School of Medicine and Biomedical Sciences, SUNY University at Buffalo, 955 Main St, Buffalo, NY 14203 USA; 16 Department of Chemistry and Physics, La Trobe Institute for Molecular Science, La Trobe University, Melbourne, VIC 3086 Australia; 17 ARC Centre of Excellence in Advanced Molecular Imaging, Department of Chemistry and Physics, La Trobe Institute for Molecular Science, La Trobe University, Melbourne, VIC 3086 Australia

**Keywords:** Structural biology, X-ray crystallography, Nanocrystallography, Microfluidics

## Abstract

Serial femtosecond crystallography (SFX) with X-ray free electron lasers (XFELs) allows structure determination of membrane proteins and time-resolved crystallography. Common liquid sample delivery continuously jets the protein crystal suspension into the path of the XFEL, wasting a vast amount of sample due to the pulsed nature of all current XFEL sources. The European XFEL (EuXFEL) delivers femtosecond (fs) X-ray pulses in trains spaced 100 ms apart whereas pulses within trains are currently separated by 889 ns. Therefore, continuous sample delivery via fast jets wastes >99% of sample. Here, we introduce a microfluidic device delivering crystal laden droplets segmented with an immiscible oil reducing sample waste and demonstrate droplet injection at the EuXFEL compatible with high pressure liquid delivery of an SFX experiment. While achieving ~60% reduction in sample waste, we determine the structure of the enzyme 3-deoxy-D-*manno*-octulosonate-8-phosphate synthase from microcrystals delivered in droplets revealing distinct structural features not previously reported.

## Abstract (web summary)

Due to the pulsed nature of X-ray free electron laser (XFEL) instruments the majority of protein crystals, which are injected using continuous jet injection techniques are wasted. Here, the authors present a microfluidic device to deliver aqueous protein crystal laden droplets segmented with an immiscible oil and demonstrate that with this device an approx. 60% reduction in sample waste was achieved for data collection of 3-deoxy-D-manno-octulosonate 8-phosphate synthase crystals at the EuXFEL.

## Introduction

The emergence of X-ray free electron lasers (XFELs) has significantly advanced X-ray crystallography in the past decade by circumventing many of the limitations of traditional goniometer-based synchrotron X-ray crystallography. Traditional macromolecular X-ray crystallography collects full data sets from irradiation of a single large crystal as it is rotated during X-ray exposure to obtain as complete a set of structure factors as possible, which subsequently support refined atomic model(s) of the subunit making up the crystalline material. Radiation-induced structural damage^1,2^ can be mitigated, but not eliminated, by data collection under cryogenic conditions, using relatively large crystals or spreading the dose over a few crystals^3^. Protein structures are successfully being determined from decreasingly small protein crystals yielding high-resolution structures at modern microfocus synchrotron beamlines. However, this is typically limited to static structures which may be altered or compromised by radiation damage^1^. Furthermore, time-resolved synchrotron macromolecular crystallography is currently limited to 100 ps time resolution and almost exclusively carried out on reversible, light-initiated reactions in large crystals. In serial femtosecond crystallography (SFX) with XFELs, each diffraction pattern is obtained before the crystal is destroyed by the intense XFEL pulse, enabling high-resolution structure determination at room temperature from radiation-sensitive samples (such as metalloproteins)^1–3^, and reaction intermediates with high time resolution^2,4–12^. SFX datasets consist of thousands of single snapshot diffraction patterns collected from microcrystals in random orientations interacting with single femtosecond-scale XFEL pulses^13^.

The European XFEL (EuXFEL) is designed to deliver trains of fs X-ray pulses with MHz repetition rates within such trains. The trains repeat at 10 Hz effectively switching off the beam >99% of the time, causing a tremendous sample waste problem if sample is delivered continuously (even if the EuXFEL runs at the full capacity of 4.5 MHz pulse repetition rate within trains in the future). Thus, a large amount of protein needs to be produced and crystallized for structure determination, creating a bottleneck where hundreds of milligrams to grams of protein are required for a complete data set^14^.

The EuXFEL MHz pulse repetition rate requires fast sample replenishing which was shown to be accomplished with high jet speeds ≥50 m/s^15,16^. Suitable injectors for crystal suspension sample injection are the gas dynamic virtual nozzle (GDVN)^17^ or liquid focusing with a double-flow focusing nozzle (DFFN), which also utilizes a GDVN^18^. The GDVN can generate high velocity jets able to replenish the sample jet between MHz pulses at the EuXFEL^16,19^, and the DFFN has achieved sufficient jet velocity in laboratory tests^20^. Sample delivery with viscous media injectors^21^ such as the lipidic cubic phase injector^22^ or fixed target approaches^23–29^ cannot keep up with the MHz repetition rate of the EuXFEL despite their advantages in reducing sample waste. Similar issues arise for the microfluidic electrokinetic sample holder (MESH)^30^ and its updated version, the concentric MESH injector (coMESH)^31^.

Droplet injection methods have the potential to overcome the limitations due to sample waste^32^, but in order to be compatible with MHz repetition rates of the EuXFEL they must achieve the fast replenishing requirements. In addition, any method for reducing sample waste, should also be compatible with time-resolved (TR) crystallography with the ultimate goal of constructing molecular movies^33^. The setup for TR-SFX for photoactivated reactions could be easily adapted to use droplet injection. However, biomolecular reactions with ligands or substrates require crystals to be mixed with reactants in solution prior to injection into the path of an XFEL. Acoustic droplet ejectors (ADEs) have demonstrated delivery of drops-on-demand for SFX with a high hit fraction^10,34–36^ usually in conjunction with a conveyor belt that transfers the droplets into the X-ray beam. However, the current ADE realizations are incompatible with the short MHz spacing of pulses at the EuXFEL, as they have been demonstrated at orders of magnitude lower frequency at available XFELs to date^10,34–36^. In addition, this approach is limited to photoinitiated TR-SFX studies and reactions involving gas-phase substrates delivered to aqueous media on millisecond time scales or above, excluding a large class of enzyme-substrate reactions. The latter limitation was however overcome with the liquid application method for time-resolved analysis recently demonstrated by Mehrabi et al.^37^, which fires pL droplets on crystals allowing biologically relevant time scales to be achieved for time-resolved studies. Piezoelectric droplet injectors^38^, in contrast, suffer from large droplet volumes increasing background scattering and are not compatible with the MHz repetition rates required by the EuXFEL.

Here, we introduce an approach to reduce sample waste in SFX experiments at the EuXFEL. It is based on the generation of sub-nL crystal suspension droplets embedded in an immiscible oil, allowing injection with a traditional GDVN. This liquid injection method is based on creating fast jets in continuous mode injection suitable for MHz crystallography and will be compatible with currently available mix-and-inject approaches^23,32^. We demonstrate droplet generation of 3-deoxy-D-*manno*-octulosonate 8-phosphate synthase (KDO8PS) crystal suspensions with a microfluidic droplet generator and show that the droplet generation frequency can be controlled by the flow rates of the aqueous and oil streams. The diffraction quality of crystals of KDO8PS is similar both when injected in aqueous droplets surrounded by oil or by continuous injection with a GDVN, with ~60% reduction in sample consumption achieved with droplet injection. The determined structure revealed new detail in a previously undefined loop region of KDO8PS, a potential target for antibiotic studies. These results from commissioning the EuXFEL advocate for future routine integration of droplet generation by segmented oil flow at other XFELs around the world. This includes the Linac Coherent Light Source (LCLS) at SLAC National Accelerator Laboratory (SLAC) operating from 30–120 Hz^39^ and other XFELs such as the Spring-8 Angstrom Compact Free Electron Laser (SACLA) pulsed at 30–60 Hz^40^, the Pohang Accelerator Laboratory XFEL (PAL-XFEL) at 60 Hz^41^ and the SwissFEL at up to 100 Hz^42^. All these instruments waste the majority of sample during continuous liquid injection^43^ which could be significantly reduced with the approach presented here.

## Results and discussion

### Droplet generator setup at EuXFEL

**Fig. 1:**
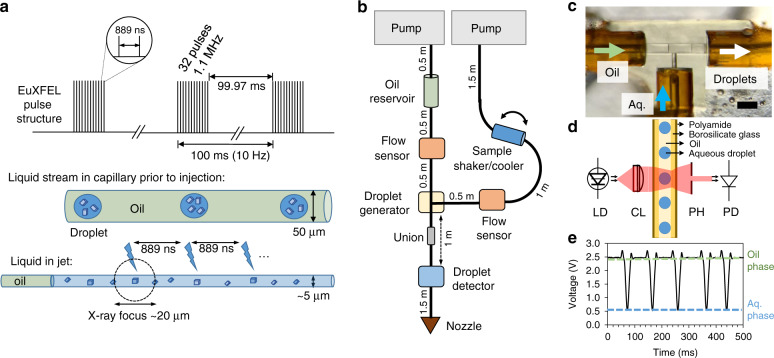
Experimental schematics. **a** Top: EuXFEL pulse structure for the early user experiment phase in which this experiment was carried out. Middle: Schematic of the segmented flow with droplets of mother liquor carrying crystals formed in an oil phase. Bottom: Schematic representation of the liquid stream leaving the GDVN, where the liquids are stretched out to form a thin jet. The stretched out droplets are larger than a pulse train of ~30 µs. **b** Schematics of the fluidic components needed to achieve segmented droplet injection at the SPB/SFX chamber (distances for each fluidic line are indicated and are approximate). **c** Brightfield optical microscopy image of an assembled droplet generator. **d** Schematic of the droplet detector and representative components: laser diode (LD), collimating lens (CL), pinhole (PH), and photodetector (PD). **e** Representative voltage plot of the optical detector for aqueous-in-oil droplets. Scale bar is 100 µm in **c**. Source data are provided as a source data file.

We present a droplet generator providing sub-nL sized droplets of aqueous crystal suspensions intersected by a continuous oil phase for experiments at the EuXFEL, which is depicted in Fig. 1. The principle consists of compartmentalizing crystal suspension in droplets segmented through an immiscible oil phase. This concept is depicted in Fig. 1, where the droplets generated in a microfluidic droplet generator are shown schematically in a capillary from which they are jetted to interact with the EuXFEL X-ray pulses. To integrate this principle in a typical SFX liquid injection setup employing a GDVN to deliver the sample in a vacuum chamber, we employed a 3D printed microfluidic droplet generator, as previously described^44^. Here, we adapted this approach for a workflow compatible with the early user experiments at the EuXFEL as depicted in Fig. 1a, which allows for macromolecular structural studies at XFELs at room temperature in a vacuum chamber. Positive pressure was applied to drive water at a constant flow rate from the HPLC pumps to the fluidic reservoirs. The HPLC pumps and flow rate sensors were remotely accessible to allow real-time adjustment of the flow rate conditions for the oil phase and crystal suspension during the experiment. The reservoir containing the crystal suspension was mounted in an anti-settling device to prevent crystal settling and was maintained at 4 °C to minimize crystal degradation^45^. Teflon pistons in the reservoirs displaced the sample or oil, and the flow rates were monitored with flow rate sensors mounted shortly after the reservoir. The crystal suspension leaving the sample reservoir flowed into the droplet generator inlet for the aqueous phase with flow rates, *Q*_aq_, ranging from 3 to 20 µL min^−1^. The oil leaving the respective reservoir entered the droplet generator at the continuous phase inlet (Fig. 1c), with oil flow rates, *Q*_oil_, ranging from 5 to 40 µL min^−1^. Thus, the total flow rate, *Q*_tot_, varied from 8 to 50 µL min^−1^, with various combinations of flow rate ratios of *Q*_oil_ to *Q*_aq_ utilized for droplet generation. Droplets exited the droplet generator through the outlet and associated capillary. Supplementary Fig. 1 shows a representative image of droplets containing crystals generated at *Q*_aq_ = 4.5 µL min^−1^ and *Q*_oil_ = 12 µL min^−1^ imaged in the capillary after the droplet generator. The capillary was connected to the GDVN by a zero dead-volume junction, assuring droplet transfer to the liquid capillary of the GDVN outside the nozzle rod (see Supplementary Fig. 1). Depending on flow rate conditions, droplet volumes ranged from ~70 to 800 pL in this study.

The capillary leaving the droplet generator was connected to another capillary passing through the droplet detector which was located about 30 cm downstream from the droplet generator. The droplet detector was crucial for this experiment as it allowed real-time feedback of the droplet generation frequency. The photodetector delivered a scalable voltage signal that allowed recording of the droplet frequency with an oscilloscope. Figure 1d shows the functioning principle of the droplet detector, taking advantage of the transmission differences between oil and aqueous solutions upon excitation at 1550 nm. Droplets were continuously observed with the droplet detector as shown in Fig. 1e. The same capillary directly served as the inlet capillary of the GDVN to inject the segmented flow into the X-ray beam. The total capillary length after the droplet generator was ~2 m to accommodate the droplet detector mounted near the top of the vacuum chamber and the full length of the nozzle rod required to insert the GDVN into the SPB/SFX vacuum chamber (Fig. 1b). At total flow rates larger than 8 µL min^−1^, we observed a stable jet with a jet diameter of about 5 µm (see Supplementary Fig. 3). We further note that aqueous droplet volumes were always large enough to form a continuous liquid element in the jet, adequate to span the time of the ~30 µs long pulse train (32 pulses repeating at 1.1 MHz in each train). The sample was hit by the X-rays in the vacuum chamber and diffraction data was collected with the AGIPD detector^46^ (see “Methods” section for details). Furthermore, we emphasize that the jets created through the glass GDVN both for the segmented flow and the continuously injected sample were fast enough for the 1.1 MHz pulse frequency within a train. Supplementary Fig. 4 shows the number of indexed patterns for each pulse ID, indicating that the indexing rate is independent of the pulse number within a train, which is a strong indication of jets fast enough for replenishing sample between pulses and in accordance with alike structures reported from MHz repeating pulses as demonstrated by Yefanov et al.^47^.

### Droplet generation frequency

The droplet generator was tested prior to the experiment using a fluorinated oil as continuous phase (10:1 PFD:PFO) and the KDO8PS mother liquor as dispersed phase to characterize the achievable droplet generation frequencies. Many factors may affect droplet generation, including the channel dimensions, liquid flow rates and velocities, liquid viscosities, and interfacial tension between the two immiscible liquids. A comprehensive equation that takes into account the aforementioned physical parameters to characterize the droplet generation frequency, $\it {\mathrm{f}}_{\mathrm{d}}$, was given by Zhang et al.^48^:

1 $$f_{\mathrm{d}} = \frac{{K \times {\mathrm{Ca}}^{{\mathrm{4/3}}}}}{W} \times \frac{{v_{\mathrm{d}}}}{{v_{{\mathrm{tot}}}}}$$

where *v*_d_ is the velocity of the dispersed (aqueous) phase, *v*_tot_ is the total velocity, Ca is the capillary number, *W* is the width of the continuous phase channel, and *K* is a pre-factor characteristic for the system, typically obtained through fitting experimental data^48^. The capillary number describes the relationship between viscous shear and interfacial forces, and equals the product of the viscosity (*η* = 13.3 mPa s) and velocity of the continuous phase divided by the interfacial tension (σ = 12 mN/m) between the two immiscible liquids. Note that the only variable term in Ca is the continuous phase velocity.

**Fig. 2:**
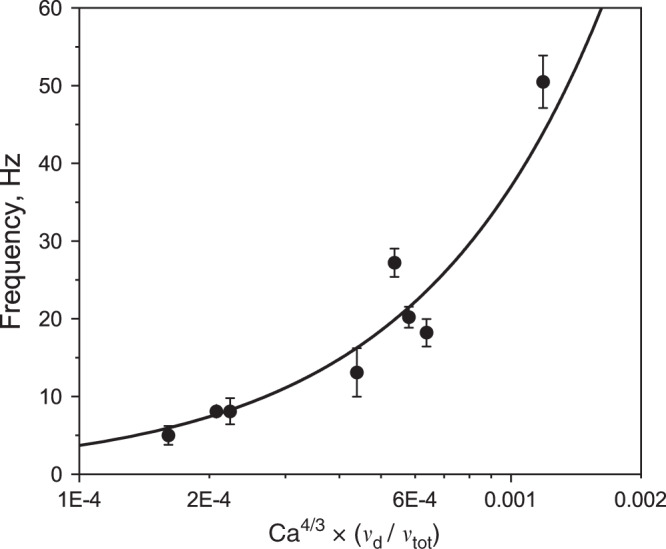
Droplet frequency characterization. The black circles represent experimentally determined data by varying *Q*_oil_ and *Q*_aq_ while maintaining a constant channel geometry. The curve is a fit of the data where *K* = 3.7 ± 0.2 m/s and the error bars represent the standard deviation. For each data point, 60–80 droplets were analyzed. Source data are provided as a source data file.

The velocities of both continuous and dispersed phases were varied by changing their flow rates, while maintaining the channel geometry, fluid viscosity, and interfacial tension constant, with the total velocity, *v*_tot_, between 10 and 20 mm/s. As illustrated in Fig. 2, the obtained droplet frequencies follow the relationship described in Eq. (1) with excellent agreement. For the flow rates tested Ca was 10^−2^ which closely corresponds to both the transient droplet generation regime between dripping and squeezing as reported by Xu et al.^49^ and Christopher et al.^50^, for which the relationship of Eq. (1) holds^48^. The best fit to the experimental data was obtained with *K* = 3.7 ± 0.2 m/s, which is close to the pre-factor 1.0 described by Zhang et al. with water as the aqueous phase^48^. We attribute the difference to variation in geometry of the T-junction, which was reported by Gupta and Kumar^51^ and Wehking et al.^52^. to affect the droplet generation frequency, as well as to the fact that Zhang et al.^48^. used pure water to generate droplets. We further note that the target 10 Hz droplet generation frequency to match the EuXFEL pulse train frequency is achievable at ${\mathrm{Ca}}^{4/3} \times \frac{{v_{\mathrm{d}}}}{{v_{{\mathrm{tot}}}}}$ = 3 × 10^−4^. In addition, droplets can be tuned to the repetition rate of other current XFEL instruments, which is shown in Supplementary Fig. 2, in accordance with Eq. (1).

### Flow rates, crystal hit fraction, and diffraction quality correlation

During the beam time experiment, we investigated the influence of the aqueous and oil flow rates on the crystal hit fraction, defined here as the average number of crystal diffraction patterns per X-ray pulse. In these experiments, the droplet generation was not synchronized with the EuXFEL pulse trains, and the X-ray beam was larger than the liquid jet diameter (see Fig. 1a bottom). If we assume a plug-flow model for the liquid jet, we expect the hit fraction *N* to follow the relation:

2 $$\left\langle N \right\rangle = \pi r_{\mathrm{j}}^2D_bnp_{{\mathrm{aq}}}$$

where $r_{\mathrm{j}}^2$ corresponds to the jet radius, *D*_b_ is the XFEL beam diameter, *n* is the number density of crystals, and $p_{{\mathrm{aq}}} = \frac{{{\mathrm{Q}}_{{\mathrm{aq}}}}}{{Q_{{\mathrm{tot}}}}}$. The jet radius is proportional to $\sqrt {Q_{{\mathrm{tot}}}}$, because the jet speed is nearly fixed by the sheath gas pressure drop^53^. Therefore, we expect the relationship $N = \pi D_{\mathrm{b}}nQ_{{\mathrm{aq}}}$, in which the hit fraction is proportional to the aqueous flow rate for a given XFEL beam diameter. This formulation may be modified to include the crystal size (assumed to be smaller than the XFEL beam) by increasing the effective XFEL beam diameter, since larger crystals will yield observable Bragg peaks at greater distances from the beam center.

**Fig. 3:**
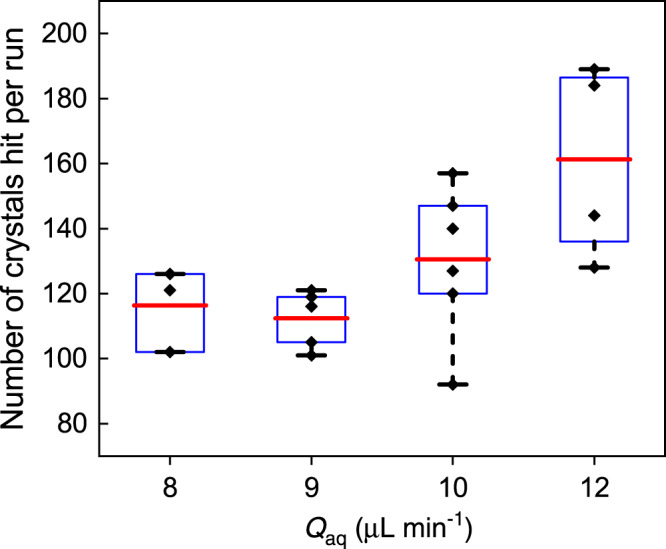
Comparison of crystals hit per run at different crystal suspension flow rates. The oil flow rate is fixed at 15 µL min^−1^, and crystal suspension flow rate is varied. The box plots show the number of crystals hit per run at each condition: rhombs represent hits per individual runs, red lines represent the mean, blue boxes contain 25th to 75th percentile, and whiskers extend to the minimum and maximum values. Source data are provided as a source data file.

In Fig. 3, several flow rate conditions are summarized underlining this behavior with the oil flow rate fixed at *Q*_oil_ = 15 µL min^−1^ whereas *Q*_aq_ is varied. The number of hits is increased as *Q*_aq_ increases, which is indicative of more crystal suspension present in the segmented jet, and thus an increased hit fraction as discussed above. Our observations were thus in agreement with expected dependencies for aqueous and oil flow rates when synchronization with the EuXFEL was not achieved.

KDO8PS diffraction data quality was further compared for crystals suspended in droplets of a segmented jet with the same sample suspended in a continuous jet (no oil present). A subset of data from 13 runs for continuous injection and 67 runs for droplet injection was selected for this analysis obtained from the same crystallization batch (1 run = 2 min). The crystal suspension was continuously injected with a flow rate of 10 μL/min for a total of 26 min (13 runs), generating an injected volume of 260 μL and resulting in 577 crystal hits. This continuously injected sample was compared to segmented flow injection of the same sample batch over the same amount of time (13 runs available for the comparison). In the segmented case, droplets were generated at different flow rate conditions, varying from 3 µL min^−1^ to 5 µL min^−1^ for *Q*_aq_, summing up to a total volume of 110 μL of crystal suspension injected over the 13 runs. The average *Q*_aq_ during this time was 4.2 µL min^−1^, resulting in 735 crystal hits. During continuous crystal suspension injection, 2.2 hits μL^−1^ were collected while 6.6 hits μL^−1^ were collected during segmented flow injection (see also Table 1).

**Table 1:** Comparison between continuous and segmented flow injection.

Condition	Continuous flow	Segmented flow (subset)	Segmented flow (all)
Collection total time (min)	26	26 (out of 134)	134
Volume injected (μL)	260	110	962
Total hits	577	735	5770
Average number of hits per μL	2.2	6.6	6.0
% indexed	53.6%	44.8%	51.9%
Average number of peaks per pattern	16.6	21.0	38.3
Average number of peaks per indexed pattern	23.1 ± 10.4	25.5 ± 17.5	35.5 ± 23.9
Average resolution (Å)	4.12	4.40	4.00

Listed are several conditions obtained for crystals in continuous flow (13 runs), for a subset in segmented flow (13 runs) and for all data in segmented flow.

Despite the observed hit fraction being small compared to previous SFX experiments at the LCLS^22,54^, it is similar to that observed in other SFX experiments conducted during the early user beam times at the EuXFEL^16,19^. This decreased hit fraction is caused by the large beam diameter of ~20 µm (compared to 2 µm at LCLS and 1 µm at SACLA for example) combined with the low pulse energy of 0.25 mJ (compared to the higher pulse energy of up to 4 mJ at LCLS). In order to measure higher resolution data, much larger crystals would be required for these experiments compared with other XFELs. These larger crystals are more prone to clogging and thereby much less crystal density can be used. Most importantly, in comparison to continuously injected sample in otherwise identical conditions, the number of crystal hits per sample volume is increased 3-fold and the sample consumption is reduced by ~60% during the segmented flow injection. Concomitantly, the diffraction quality is comparable for both cases with regard to average resolution, highest resolution, average number of peaks per pattern for all hits, and average number of peaks per indexed pattern, as listed in Table 1.

Overall, KDO8PS crystal suspension was injected using segmented flow for a total of 134 min summed over the entire beam time, where data collection was possible. Within this time, the crystal suspension flow rate ranged between 3 and 12 µL min^−1^ injecting a total volume of 962 µL of suspension. An average of 6.0 hits µL^−1^ of sample was obtained with an average resolution of 4 Å. This clearly indicates that the oil phase and droplet generation procedure did not impact crystal quality and did not affect the data quality for structure determination. To further substantiate this finding, we performed a diffuse scattering analysis combining radial profiles for injection conditions corresponding to co-flow of aqueous crystal suspension and oil in parallel, as well as segmented droplet injection (Supplementary Fig. 7). From this comparison, it can be observed that an oil ring prevails in the diffuse scattering for the co-flowing case in almost all of the images, whereas for the droplet injection, only very few select images displayed this oil scattering ring. We can therefore conclude that the majority of crystals were injected in an aqueous phase, and not “mixed” with the oil slugs. The few instances of oil apparent for the diffuse scattering analysis can be attributed to hitting the start or end of the droplet where both aqueous and oil phase are present. The sparsity of the oil scattering for the droplet injection further demonstrates the accurate phase overlap of the sample droplets with the X-ray pulse train. A representative diffraction pattern for KDO8PS is shown in Supplementary Fig. 8.

### SFX structure of KDO8PS

The SFX structure of KDO8PS was solved based on all collected data where droplet formation was confirmed via the in-line droplet detector. The mean of unit cell constant distributions (Supplementary Fig. 3) were calculated to be a = b = c = 118.4 Å and α = β = γ = 90° by cell_explorer, part of the CrystFEL package^55^. Of the 37,000 patterns classified as hits, 16,777 could be indexed and 15,777 patterns were included in the final set of merged reflections (1000 patterns were rejected by partialator during the merging) and used for structure determination. Detected diffraction peaks reached a resolution of 2.8 Å (see Supplementary Fig. 5). An overview of the data collection and processing parameters and statistics is presented in Supplementary Tables 1 and 2. Further details of the subset of all confirmed segmented flow data (134 min) are shown in Supplementary Table 1.

**Fig. 4:**
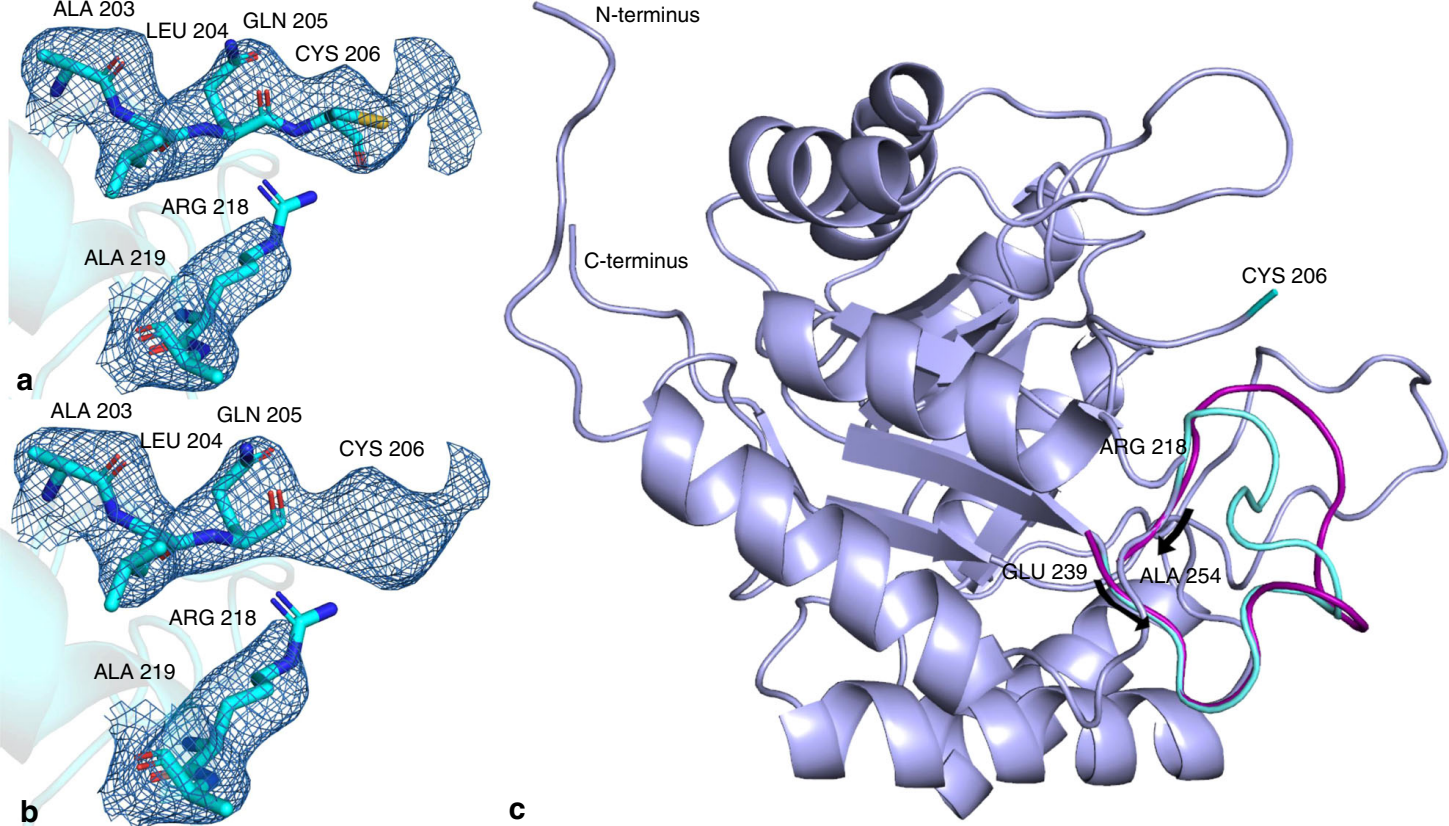
Differences between our refined model and the pdb entry 1X8F used as the search model. **a** and **b** 2Fo-Fc electron density map contoured at 1.0 σ in the undefined loop region ranging from amino acids 206 through 218 missing in the search model 1X8F. Residual electron density can clearly be seen extending beyond GLN−205 in **b** and a cysteine was built into position 206 and refined (**a**). **c** Cartoon representation of the refined model superimposed on PDB entry 1X8F. The different loop conformation (residue range 246-251) is highlighted in cyan (refined model, deposited to the PDB accession code 6U57) and magenta (search model, PDB entry 1X8F). The diffraction patterns for 6U57 are available on the Coherent X-ray Imaging Data Bank (https://cxidb.org) with entry ID 152.

It has been reported previously^56,57^ that refinement of the KDO8PS structure is particularly difficult due to the inherent heterogeneity of the arrangement of monomers in the KDO8PS tetramer, and three 2-fold symmetry axes intersecting in the center of the tetramer. This was observed for the data presented here, with the structure refining to a final R_work_/R_free_ of 18.6/24.9 under our highly constrained refinement protocol. Such values are also in good agreement with the structure published previously by Vainer et al.^56^ which was also solved by applying molecular replacement, but at cryogenic temperatures at a synchrotron radiation source. Furthermore, the structures reported by both Radaev et al.^57^ and Vainer et al.^56^ have a missing, unstructured loop region (residues AA-206 to AA-217), which is believed to be part of the active site of the protein. In the structure presented here, residue CYS-206 showed clear electron density and was therefore included in the model (Fig. 4a, b). A further deviation from the previously published model by Vainer et al.^56^ was found in the loop region ranging from residue 246 through to 251, which was modified from the 1X8F search model in accordance with the clear deviation present in the electron density (Fig. 4c). This loop is completely omitted from the structure published by Radaev et al.^57^, indicating that it is a highly flexible loop. KDO8PS is a flexible protein, indicated by the fact that it shows severe conformational changes upon ligand binding^56^. Our structure represents the apo protein structure at room temperature, wheras the published structures were determined under cryogenic conditions, where cryo-protectant was added^58,59^. Both, the addition of the cryo-protectant, as well as the freezing process can induce conformational changes or select a subset of conformations present at room temperature. Examples for changes in room temperature XFEL and cryogenic crystallography structures were also observed for the serotonin receptor^60^.

We have presented an effective way to mitigate the sample waste inherent to continuous liquid sample injection for SFX at pulsed XFEL sources, a challenge exacerbated by the unique pulse structure of the EuXFEL. We demonstrated that a 3D printed microfluidic droplet generator can be integrated into the workflow of an SFX experiment at the SPB/SFX instrument at the EuXFEL and have successfully injected droplets containing protein crystals into the MHz repetition rate X-ray beam. In comparison to continuous injection from a GDVN, segmented flow injection resulted in a higher number of crystal hits per volume, while maintaining the same quality of SFX data.

A room temperature structure of KDO8PS was determined from microcrystals delivered with the segmented flow generator. The electron density revealed differing detail compared to previously-reported KDO8PS structures determined at cryogenic temperatures^56,57^, such as the CYS-206 residue and a different loop conformation in the residue range 246–251 (Fig. 4c). Despite the slightly lower resolution of the room temperature structure, the fact that these differences emerged in such a flexible structure are an indicator that the data is of good quality. In the future, we also plan time-resolved SFX studies on KDO8PS to investigate if the here observed structural changes relate to functionality involved in the catalysis mechanism of the enzyme. The results outlined in this article paint a promising future for segmented flow sample delivery mitigating the sample waste problem of the unique pulse structure of the EuXFEL. With droplets synchronized with the frequency and phase of the X-ray pulses, we expect a further reduction in the volume of sample consumed (potentially down to 1% of continuous injection at the EuXFEL), allowing the exploration of new crystal samples that do not crystallize readily in large volumes and accomplishing structure determination at MHz XFELs in the order of minutes^61^. Efforts to synchronize droplets in a 3D printed droplet generator via electrical triggering are currently being explored^62^. Furthermore, the droplet generation frequency is tunable, presenting a promising future for segmented flow sample delivery of scarce, hard-to-crystallize samples and more efficient data collection for studying macromolecular dynamics at other current and future XFELs. The presented segmented flow approach is also compatible with mix-and-inject time resolved serial crystallography, such as demonstrated by Ishigami et al.^63^, and has the potential to reach ms time resolution as recently demonstrated by Knoska et al. with ultracompact 3D microfluidics^20^.

## Methods

### Chemicals and materials

IP-S photoresist was purchased from Nanoscribe GmbH (Germany); SU-8 developer, from Microchem (USA); Novec 1720, from 3 M (USA); isopropyl alcohol, from VWR Analytical (USA); epoxy (#04001) from Hardman Inc. (USA); perfluorodecalin (PFD), from Sigma-Aldrich (USA); 1H,1H,2H,2H-perfluorooctanol (PFO), from Alfa Aesar Co. Inc. (USA); KDO8PS genes, from GenScript Inc. (USA); *E. Coli*, from New England Biolabs (USA); Sigma Fast tablets, β-mercaptoethanol, Tris, HCl, ethylenediaminetetraacetic acid (EDTA), protamine sulfate, KCl, and protease inhibitor cocktail, from Sigma-Aldrich (USA); and poly(ethylene glycol) 5000 methyl ether (PEG5000 MME), from Hampton Research (USA). Fused silica capillaries were obtained from Molex LLC (USA); tubing and capillary union connectors, from IDEX Health and Science LLC (USA); PicoClear unions, from New Objective, Inc. (USA); PEEK tubing, from Zeus (USA); double sided tape, from 3 M (USA); and 10 kDa cutoff filters, from Centricon, Millipore (USA).

### Sample preparation

The wild-type KDO8PS gene (GenBank accession number NC_000913) was purchased from GenScript as a synthetic gene in the pET-23d plasmid. The complete and annotated vector map, plasmid DNA and protein sequences are detailed in Supplementary Figs. 9–11. This plasmid was subsequently transformed into LEMO21(DE3) competent *E. coli* cells and expressed according to the protocols described in refs. ^58,64^ as follows. The protein was overexpressed in Lemo21 (DE3) *E. coli* cells under the control of a T7 promoter. LB media containing ampicillin and chloramphenicol was inoculated with the culture and vigorously shaken at 37 °C until an A600 = 0.6–0.8 was reached and then 0.2 mM isopropyl-1-thio-ß-D-galactopyranoside was added. After 4 h of incubation at 37 °C the cells were harvested by centrifugation at 8000 rcf for 10 min at 4 °C and then frozen at −80 °C. The frozen pellet was re-suspended in a buffer containing 20 mM Tris pH 7.3, 300 mM potassium chloride, 1 SIGMAFAST protease inhibitor cocktail tablet (EDTA-free) (Sigma), and 10 mg/1 g cell pellet lyophilized lysozyme. After suspension, the cells were incubated in the lysis buffer for 30 min at room temperature followed by incubation on ice for 20 min. The cells were then broken using a sonicator with a microtip at 50% power, 10 s pulses for 6 cycles totaling 1 min with a 30 s incubation on ice in between each cycle. The cells were then centrifuged at 24,000×*g* for 30 min. A protamine sulfate precipitation was then performed on the supernatant. For this step a 2.2% protamine sulfate, 20 mM Tris pH 5, 300 mM potassium chloride, and 1 SIGMAFAST protease inhibitor cocktail tablet (EDTA-free) solution was added to achieve a final concentration of 0.26%. The precipitation was carried out for 15 min on ice and then the solution was centrifuged for 30 min at 48,000×*g*. The supernatant was then dialyzed against 5 mM potassium phosphate pH 7.3, 75 mM potassium chloride. One buffer exchange was carried out after 4 h. The dialyzed enzyme preparation was then centrifuged at 7000×*g* at 4 °C for 10 min to remove precipitated proteins.

After centrifugation, the supernatant was filtered using a 0.22 μm syringe filter. Aliquots were injected into an anion exchange column (900 mm × 16 mm, DEAE-sepharose) equilibrated with 20 mM Tris-HCl pH = 7.3, 75 mM KCl, and 2 mM β-mercaptoethanol. The salt concentration was increased to 125 mM KCl to elute KDO8PS fractions. Purity was determinded by sodium dodecyl sulfate polyacrylamide gel electrophoresis (SDS-PAGE) and a thiobarbituric acid assay was used to confirm KDO8PS functional activity. In addition, mass spectrometry by MALDI-TOF/TOF and dynamic light scattering (DLS) were used to confirm the identity of KDO8PS and to characterize the monodispersity of the sample prior to crystallization. Pure fractions of KDO8PS were pooled and concentrated with a 10 kDa cutoff centrifugal filter until an absorbance value of A_205_ = 0.645 was achieved. The concentrated solution was frozen and stored at −80 °C until crystallization. KDO8PS microcrystals were grown using a stirred batch method^65^ where the final concentration of KDO8PS was adjusted to 8.75–12 mg/mL mL in a buffer containing 20 mM Tris-HCl, pH 7.4 150 mM KCl. Crystallization was induced by dropwise addition of 16–20% w/v PEG5000 MME from a 50% stock solution in water. The final crystals for sample delivery were grown in multiple batches of 600 µL each in 1.5 mL reaction vessels at room temperature (25˚C). Under optimized conditions for this experiment, 300 µL of 20% PEGMME 5000 (from a 50% stock solution in water) were dropped into 300 µL of KDO8PS solution at 21 mg/mL, while stirring with a small thin stir bar (0.5 mm × 5 mm). The drops were added using a 1000 µL pipette over a time period of approximately 2 min. The crystallization solution was then incubated at room temperature for 1.5 h without stirring. The crystals settle during this time. They were resuspended in the supernatant of the crystallization experiment directly before sample delivery at a volume ratio of pellet to supernatant of 1 to 10 leading to a crystal density of approximately 5 × 10^9^ crystals/mL as estimated by counting crystals in a cell counting chamber. Crystal batches were monitored and inspected under a stereomicroscope and characterized by dynamic light scattering to select the most uniform crystals for desired size and size homogeneity. Crystal batches used for this experiment consisted of uniform crystals of the size range between 8 and 10 µm. For the data reported in Table 1, the crystal suspension from the same crystallization condition was used both in the continuous flow injection, as well as the segemented injection case for the comparison of 26 runs.

### Droplet generator fabrication

The continuous oil phase was prepared by mixing PFD and PFO in a 10:1 v/v ratio. Droplet generator devices were fabricated by two photon polymerization (2pp) 3D printing^44^. Briefly, models were designed in AutoCAD (AutoDesk, USA) and 3D printed using IP-S photoresist and the Photonic Professional GT 3D printer (Nanoscribe GmbH, Germany). Printing was accomplished in solid mode using dip-in laser lithography 2pp. Once printed, devices were developed in SU-8 developer and rinsed in isopropyl alcohol. Each device was then immobilized on a glass slide with tape, fused silica capillaries with polished ends were inserted and glued into the device inlets and outlet with epoxy. Lastly, the device, GDVN, and capillaries were surface treated filled with Novec 1720 and the devices were placed in an oven at 150 °C overnight to remove excess solvent^44^. The fused silica capillary outer diameter (OD) was 360 µm and the inner diameter (ID) varied (either 50, 75, or 100 µm). The T-junction was defined by the intersection of a 100 µm × 75 µm × 650 µm rectangular channel and a 50 µm diameter cylindrical channel (see Fig. 1c and Supplementary Fig. 1).

### Fluidic set-up

Positive pressure was applied using HPLC pumps (LC-20AD from Shimadzu Co., Japan) to initiate and control the fluid flow. Each HPLC pump delivered water to a custom made or commercial reservoir containing a piston from which either crystal suspension or oil phase was dispensed^59^. Pressures were adjusted to provide the intended flow rates, with pressures ranging from ~200 psi to 800 psi, depending on the exact length of fluidic tubing employed, the crystal sample injected and other factors influencing the pressure and thus flow rates. For XFEL experiments, the crystal suspension reservoir was mounted on a rotating anti-settling device with temperature control at 4 °C^59^. PEEK tubing and fluidic connections were used to connect components upstream of the droplet generator device, while fused silica capillaries and PicoClear unions were used to connect the droplet generator to the downstream glass GDVN. GDVNs were similar to those used by Gisriel et al.^66^. The distance from the droplet generator to the tip of the GDVN was about 2.5 m. Liquid flow meters SLI-0430 and SLG-0075 (Sensirion, Switzerland) were used to monitor the flow rates after the reservoirs (see Fig. 1b for a schematic of the entire fluidic set-up and Supplementary Fig. 1 for additional details).

Using this setup, droplets were generated at the T-junction. The droplet volumes were large enough to cover the full duration of a pulse train (~30 µs) as specified below in section XFEL instrument setup and Fig. 1a. The droplet volumes were in the range of ~70–800 pL depending on droplet frequency defined by the flow rate of aqueous and oil phases. Thus, with the used total flow rates ranging between 8 and 45 µL min^−1^, droplets were present in the jet for about 100–500 µs, well above the duration of a pulse train. In addition, when flow rate conditions were changed, an equilibration of about 10 min was allowed to stabilize the pressure in the system and consequently the droplet frequency. Flow rate stability was additionally monitored through the flow rate sensors.

### Droplet detection

All components were purchased from Thorlabs, USA, unless stated otherwise. Briefly, a 1550 nm 5 mW laser beam (a L1550P5DFB laser diode and a LTC56B Controller Kit) was transmitted across the fused silica capillary connecting the T-junction droplet generator with the GDVN, and the transmitted light was detected with an amplified photodetector (PDA20CS). A collimating lens (C230TMD-C) on a kinematic mount (KC1-T), a fused silica capillary custom built holder, and a 200 μm pinhole were aligned between the laser diode and the detector using x–y translators (SCPO5T) within a 30 mm cage system. The signal (Fig. 1e) was displayed and recorded on an oscilloscope (TDS 2024, Tektronix). Droplet experiments outside of an XFEL facility were additionally monitored with brightfield optical microscopy (IX71, Olympus, USA) and a Photron high speed camera (FASTCAM SA4, Japan). MicroManager (ver 1.4.22, UCSF, USA) and ImageJ (ver 1.48 v, NIH, USA) software were used for image acquisition, processing, and analysis, and Origin (OriginLab Corp., USA) was used to generate plots.

### XFEL instrument setup

Experiments were conducted at the EuXFEL (Schenefeld, Germany) upstream interaction region of the SPB/SFX instrument^67^ during the beamtime P2042. The pulse structure of the XFEL was composed of 10 Hz trains, with 32 pulses per train, see Fig. 1a. The repetition rate in the train was 1.1 MHz or  889 ns between pulses. The pulse duration was ≤100 fs^68^ with a photon energy of 9.31 keV. The beam was focused with compound refractive lenses to a beam size of 15 × 20 µm^2^. The average pulse energy was 280 µJ. Diffraction data was collected using an AGIPD 1 Mpx detector^46^ at a detector distance of 173.5 mm^69,70^. For data collection, diffraction data was not used for the first two pulses in the train as these were calibration pulses. Data recording started with pulse 3 and every second consecutive pulse was then used, such that 15 diffraction patterns were recorded for each pulse train. The GDVN was attached to the end of a nozzle rod (~1 m in length) that was inserted through an airlock system into the 10^−5^ mbar vacuum chamber. Helium gas pressure of 150 psi was used to operate the GDVN and was controlled by GP1 electronic pressure regulators (Equilibar, USA).

### Diffuse scattering analysis

In order to investigate whether the crystals hit by the X-ray pulses for the droplet injection approach were in the aqueous or the oil phases, we conducted a basic background analysis of the diffuse scattering for a single run. Two central panels of the AGIPD detector (panels 3 & 4 according to CrystFEL convention) were selected for this analysis. For an entire run of (background corrected) hits found by Cheetah, the pixel values of each panel for every hit were projected horizontally, resulting in an unscaled diffuse scattering profile. This analysis was performed for both a 2 min droplet injection run, as well as a 2 min run where oil and crystal suspension were co-flowing (Supplementary Fig. 7).

### Data processing

The KDO8PS diffraction patterns collected were identified and calibrated using Cheetah^71^. For hit-finding, a minimum of 2 connected pixels with a count above a threshold of 500 ADU with a minimum signal-to-noise ratio of 8 was considered a peak, and an image containing at least 10 such peaks was classified as a hit. From the ~4,100,000 images collected, 37,000 were classified as hits, which corresponds to an average hit rate of 0.9%.

Approximately 46% of the identified hits could be indexed and the Bragg reflections integrated using the software package CrystFEL (version 0.8.0)^55^, based on the peak locations found by Cheetah. Indexing was performed by CrystFEL’s indexamajig sequentially trying XGANDALF^72^, DirAx^73^, MOSFLM^74^ and XDS^75^ requiring a cubic body-centered lattice and unit cell parameters of a = b = c = 118 Å and α=β=γ=90° (Supplementary Fig. 5). The indexamajig integration radii were set to 2, 3, 4 pixels and indexing solutions were checked by ensuring that they accounted for at least 50% of the observed peaks (option “check-peaks”).

The indexed patterns were further processed with ambigator^76^ to resolve the indexing ambiguity inherent to the cubic space group I23 for serially collected data. The number of correlation coefficients per crystal was limited to 1000 for speed, and 10 passes of ambiguity resolution were made over all crystals. This resulted in 8315 indexing assignments being changed, corresponding to roughly 50% of the data as expected. The indexed reflections were subsequently scaled and merged using partialator^77^, applying the unity model (without partiality modeling) over 3 iterations. The intensities were converted to structure factor moduli using truncate (from the CCP4 suite^78^), and a fraction of 0.1 reflections were included in the generated R_free_ set. The L-test implemented in truncate was used to ascertain successful de-twinning of the data (Supplementary Fig. 6).

### Structure solution and refinement

Phasing was performed using molecular replacement with Phaser^79^ of the CCP4i program suite^78^ using the PDB code 1X8F^56^ as search model. The obtained model was refined using alternate cycles of automated refinement with REFMAC^80^ and manual inspection was performed with COOT^81^. The final refined structure was assessed using the web server PDB-REDO^82^, which indicated that the R_free_ value is biased. The R_free_ value reported in the results is therefore the unbiased R_free_ value, as defined by PDB-REDO^83^. All Figures of the protein structure presented in this manuscript were generated in PYMOL^84^. The final refined structure was validated using the wwwPDB Validation Service and submitted to the Protein Data Bank for deposition with PDB ID 6U57.

### Reporting summary

Further information on experimental design is available in the Nature Research Reporting Summary linked to this paper.

## Supplementary information

Supplementary Information

Reporting summary

## Data Availability

The protein model and the associated structure factors have been deposited in the Protein Data Bank with accession code 6U57. Furthermore, the diffraction patterns have been uploaded to the Coherent X-ray Imaging Data Bank (https://cxidb.org) along with the stream file outlining the indexing parameters for the individually indexed patterns under entry ID 152. Other data are available from the corresponding author upon reasonable request. Source data are provided with this paper.

## Source data

Source Data

## References

[1] Bowman SE, Bridwell-Rabb J, Drennan CL (2016). Metalloprotein crystallography: more than a structure. Acc. Chem. Res..

[2] Chapman HN (2011). Femtosecond X-ray protein nanocrystallography. Nature.

[3] Andersson R (2017). Serial femtosecond crystallography structure of cytochrome c oxidase at room temperature. Sci. Rep..

[4] Pande K (2016). Femtosecond structural dynamics drives the trans/cis isomerization in photoactive yellow protein. Science.

[5] Coquelle N (2018). Chromophore twisting in the excited state of a photoswitchable fluorescent protein captured by time-resolved serial femtosecond crystallography. Nat. Chem..

[6] Barends TR (2015). Direct observation of ultrafast collective motions in CO myoglobin upon ligand dissociation. Science.

[7] Suga M (2017). Light-induced structural changes and the site of O=O bond formation in PSII caught by XFEL. Nature.

[8] Wickstrand C (2019). Bacteriorhodopsin: structural insights revealed using X-ray lasers and synchrotron radiation. Annu. Rev. Biochem..

[9] Nass Kovacs G (2019). Three-dimensional view of ultrafast dynamics in photoexcited bacteriorhodopsin. Nat. Commun..

[10] Kern J (2018). Structures of the intermediates of Kok’s photosynthetic water oxidation clock. Nature.

[11] Nogly P (2018). Retinal isomerization in bacteriorhodopsin captured by a femtosecond x-ray laser. Science.

[12] Nango E (2016). A three-dimensional movie of structural changes in bacteriorhodopsin. Science.

[13] Johansson LC, Stauch B, Ishchenko A, Cherezov V (2017). A bright future for serial femtosecond crystallography with XFELs. Trends Biochem. Sci..

[14] Martin-Garcia JM, Conrad CE, Coe J, Roy-Chowdhury S, Fromme P (2016). Serial femtosecond crystallography: a revolution in structural biology. Arch. Biochem. Biophys..

[15] Wiedorn MO (2018). Rapid sample delivery for megahertz serial crystallography at X-ray FELs. IUCrJ.

[16] Wiedorn MO (2018). Megahertz serial crystallography. Nat. Commun..

[17] Weierstall U, Spence JC, Doak RB (2012). Injector for scattering measurements on fully solvated biospecies. Rev. Sci. Instrum..

[18] Oberthuer D (2017). Double-flow focused liquid injector for efficient serial femtosecond crystallography. Sci. Rep..

[19] Grunbein ML (2018). Megahertz data collection from protein microcrystals at an X-ray free-electron laser. Nat. Commun..

[20] Knoška J (2020). Ultracompact 3D microfluidics for time-resolved structural biology. Nat. Commun..

[21] Conrad CE (2015). A novel inert crystal delivery medium for serial femtosecond crystallography. IUCrJ.

[22] Weierstall U (2014). Lipidic cubic phase injector facilitates membrane protein serial femtosecond crystallography. Nat. Commun..

[23] Echelmeier A, Sonker M, Ros A (2019). Microfluidic sample delivery for serial crystallography using XFELs. Anal. Bioanal. Chem..

[24] Hunter MS (2014). Fixed-target protein serial microcrystallography with an x-ray free electron laser. Sci. Rep..

[25] Roedig, P. et al. A micro-patterned silicon chip as sample holder for macromolecular crystallography experiments with minimal background scattering. *Sci. Rep.* **5**, 10.1038/srep10451 (2015). 10.1038/srep10451 PMC4448500 26022615

[26] Sherrell DA (2015). A modular and compact portable mini‐endstation for high‐precision, high‐speed fixed target serial crystallography at FEL and synchrotron sources. J. Synchrotron Radiat..

[27] Roedig P (2016). Room-temperature macromolecular crystallography using a micro-patterned silicon chip with minimal background scattering. J. Appl. Crystallogr..

[28] Oghbaey S (2016). Fixed target combined with spectral mapping: approaching 100% hit rates for serial crystallography. Acta Crystallogr. Sect. D..

[29] Owen RL (2017). Low‐dose fixed‐target serial synchrotron crystallography. Acta Crystallogr. Sect. D..

[30] Sierra RG (2012). Nanoflow electrospinning serial femtosecond crystallography. Acta Crystallogr. Sect. D..

[31] Sierra RG (2016). Concentric-flow electrokinetic injector enables serial crystallography of ribosome and photosystem II. Nat. Methods.

[32] Cheng RKY (2020). Towards an optimal sample delivery method for serial crystallography at XFEL. Crystals.

[33] Srajer V, Schmidt M (2017). Watching proteins function with time-resolved X-ray crystallography. J. Phys. D. Appl Phys..

[34] Roessler CG (2016). Acoustic injectors for drop-on-demand serial femtosecond crystallography. Structure.

[35] Fuller FD (2017). Drop-on-demand sample delivery for studying biocatalysts in action at X-ray free-electron lasers. Nat. Methods.

[36] Young DI (2016). Structure of photosystem II and substrate binding at room temperature. Nature.

[37] Mehrabi P (2019). Liquid application method for time-resolved analyses by serial synchrotron crystallography. Nat. Methods.

[38] Mafune F (2016). Microcrystal delivery by pulsed liquid droplet for serial femtosecond crystallography. Acta Crystallogr. D..

[39] Emma P (2010). First lasing and operation of an angstrom-wavelength free-electron laser. Nat. Photon..

[40] Tono, K. et al. Beamline, experimental stations and photon beam diagnostics for the hard x-ray free electron laser of SACLA. *New J. Phys.* **15**, 10.1088/1367-2630/15/8/083035 (2013).

[41] Park J, Kim S, Nam KH, Kim B, Ko IS (2016). Current status of the CXI beamline at the PAL-XFEL. J. Korean Phys. Soc..

[42] Milne CJ (2017). SwissFEL: the Swiss X-ray free electron laser. Appl. Sci..

[43] Weierstall U (2014). Liquid sample delivery techniques for serial femtosecond crystallography. Philos. Trans. R. Soc. Lond. B Biol. Sci..

[44] Echelmeier A (2019). 3D printed droplet generation devices for serial femtosecond crystallography enabled by surface coating. J. Appl. Crystallogr.

[45] Schulz J (2019). A versatile liquid-jet setup for the European XFEL. J. Synchrotron Radiat..

[46] Allahgholi A (2019). The adaptive gain integrating pixel detector at the European XFEL. J. Synchrotron Radiat..

[47] Yefanov O (2019). Evaluation of serial crystallographic structure determination within megahertz pulse trains. Struct. Dyn..

[48] Zhang SH, Guivier-Curien C, Veesler S, Candoni N (2015). Prediction of sizes and frequencies of nanoliter-sized droplets in cylindrical T-junction microfluidics. Chem. Eng. Sci..

[49] Xu JH, Li SW, Tan J, Luo GS (2008). Correlations of droplet formation in T-junction microfluidic devices: from squeezing to dripping. Microfluid. Nanofluid..

[50] Christopher GF, Noharuddin NN, Taylor JA, Anna SL (2008). Experimental observations of the squeezing-to-dripping transition in T-shaped microfluidic junctions. Phys. Rev. E Stat. Nonlin. Soft Matter Phys..

[51] Gupta A, Kumar R (2010). Flow regime transition at high capillary numbers in a microfluidic T-junction: viscosity contrast and geometry effect. Phys. Fluids.

[52] Wehking JD, Gabany M, Chew L, Kumar R (2014). Effects of viscosity, interfacial tension, and flow geometry on droplet formation in a microfluidic T-junction. Microfluid. Nanofluid..

[53] Gañán-Calvo AM (1998). Generation of steady liquid microthreads and micron-sized monodisperse sprays in gas streams. Phys. Rev. Lett..

[54] Jonsson HO, Caleman C, Andreasson J, Timneanu N (2017). Hit detection in serial femtosecond crystallography using X-ray spectroscopy of plasma emission. IUCrJ.

[55] White TA (2012). CrystFEL: a software suite for snapshot serial crystallography. J. Appl. Crystallogr..

[56] Vainer R, Belakhov V, Rabkin E, Baasov T, Adir N (2005). Crystal structures of Escherichia coli KDO8P synthase complexes reveal the source of catalytic irreversibility. J. Mol. Biol..

[57] Radaev S, Dastidar P, Patel M, Woodard RW, Gatti DL (2000). Structure and mechanism of 3-deoxy-D-manno-octulosonate 8-phosphate synthase. J. Biol. Chem..

[58] Coe, J. Life in motion: visualizing biomacromolecules by time-resolved serial femtosecond crystallography. In: *Center for Applied Structural Discovery.* The Biodesign Institute (Arizona State University, 2018).

[59] Lomb L (2012). An anti-settling sample delivery instrument for serial femtosecond crystallography. J. Appl. Crystallogr..

[60] Liu W (2013). Serial femtosecond crystallography of G protein-coupled receptors. Science.

[61] Pandey S (2020). Time-resolved serial femtosecond crystallography at the European XFEL. Nat. Methods.

[62] Kim D (2019). Electric triggering for enhanced control of droplet generation. Anal. Chem..

[63] Ishigami I (2019). Snapshot of an oxygen intermediate in the catalytic reaction of cytochrome c oxidase. Proc. Natl Acad. Sci. USA.

[64] Conrad, C. E. *Overcoming Barriers in Structural Biology Through Method Development of Serial Crystallography*. (Arizona State University, 2016).

[65] Kupitz C (2014). Microcrystallization techniques for serial femtosecond crystallography using photosystem II from Thermosynechococcus elongatus as a model system. Philos. Trans. R. Soc. Lond. B Biol. Sci..

[66] Gisriel C (2019). Membrane protein megahertz crystallography at the European XFEL. Nat. Commun..

[67] Mancuso AP (2019). The single particles, clusters and biomolecules and serial femtosecond crystallography instrument of the European XFEL: initial installation. J. Synchrotron Radiat..

[68] Geloni G (2010). Coherence properties of the European XFEL. N. J. Phys..

[69] Boukhelef, D., Szuba, J., Wrona, K. & Youngman, C. Software development for high speed data recording and processing. CALEPCS2013 (2013).

[70] Fangohr, H. et al. Data Analysis support in Karabo at European XFEL. In: *16th International Conference on Accelerator and Large Experimental Control Systems*) (JACoW Publishing, 2017).

[71] Barty A (2014). Cheetah: software for high-throughput reduction and analysis of serial femtosecond X-ray diffraction data. J. Appl .Crystallogr..

[72] Gevorkov, Y. et al. XGANDALF–extended gradient descent algorithm for lattice finding. *Acta Crystallogr. Sect. A* **74**, 694–704 (2019). 10.1107/S2053273319010593 PMC6718201 31475914

[73] Duisenberg AJM (1992). Indexing in single-crystal diffractometry with an obstinate list of reflections. J. Appl. Crystallogr..

[74] Powell HR, Johnson O, Leslie AG (2013). Autoindexing diffraction images with iMosflm. Acta Crystallogr. D. Biol. Crystallogr..

[75] Kabsch W (2010). Xds. Acta Crystallogr. D. Biol. Crystallogr..

[76] White TA (2016). Recent developments in CrystFEL. J. Appl. Crystallogr..

[77] White TA (2019). Processing serial crystallography data with CrystFEL: a step-by-step guide. Acta Crystallogr. D. Struct. Biol..

[78] Winn MD (2011). Overview of the CCP4 suite and current developments. Acta Crystallogr. D. Biol. Crystallogr..

[79] McCoy AJ (2007). Phaser crystallographic software. J. Appl. Crystallogr..

[80] Murshudov GN (2011). REFMAC5 for the refinement of macromolecular crystal structures. Acta Crystallogr. D. Biol. Crystallogr..

[81] Emsley P, Lohkamp B, Scott WG, Cowtan K (2010). Features and development of Coot. Acta Crystallogr. D. Biol. Crystallogr..

[82] Joosten RP, Long F, Murshudov GN, Perrakis A (2014). The PDB_REDO server for macromolecular structure model optimization. IUCrJ.

[83] Joosten RP, Joosten K, Murshudov GN, Perrakis A (2012). PDB_REDO: constructive validation, more than just looking for errors. Acta Crystallogr. D. Biol. Crystallogr..

[84] Schrödinger, L. L. C. The PyMOL Molecular Graphics System, Version~1.8. (2015).

