# Supplementary material for: Segmented flow generator for serial crystallography at the European X-ray free electron laser
Source: Nat Commun. 2020 Sep 9;11:4511. doi: 10.1038/s41467-020-18156-7 (PMC7481229; doi:10.1038/s41467-020-18156-7)
Supplement: Supplementary file 1 — Supplementary Information [file 41467_2020_18156_MOESM1_ESM.pdf]

Supplementary Information

Austin Echelmeier, Jorvani Cruz Villarreal et al.

Supplementary Table 1: Data Collection Statistics

Values for the outer shell are given in parentheses.

	All data	Droplet Data Subset
Diffraction source	European XFEL, SPB/SFX	European XFEL, SPB/SFX
Wavelength (Å)	1.33	1.33
Temperature (K)	293 K	293 K
Detector	AGIPD	AGIPD
Crystal-detector distance (mm)	173.5	173.5
No. of indexed crystal patterns	15777	4763
Exposure time per image (fs)	100	100
Space group	I23	I23
a, *b*, *c* (Å)	118.4, 118.4, 118.4	118.4, 118.4, 118.4
α, β, γ (°)	90.0, 90.0, 90.0	90.0, 90.0, 90.0
Resolution range (Å)	48.0-2.80 (2.90-2.80)	48.0-2.80 (2.90-2.80)
Total No. of reflections	1 268 974 (99 319)	354 193 (27 745)
No. of unique reflections	6888 (674)	6888 (674)
Completeness (%)	100 (100)	100 (100)
Multiplicity	184.2 (147.4)	51.4 (41.2)
$\langle I/\sigma(I)\rangle$ #	4.61 (0.95) #	2.64 (0.56)
CC*	0.9953 (0.7003)	0.9771 (0.4459)
R_{split}	14.3 (121.2)	29.4 (209.8)
Overall *B* factor from Wilson plot (Å^2)	84.6	85.7

# It is common practice in serially collected diffraction data to include data down to a CC of 0.5, which commonly gives an $I/\sigma(I)<2.0$ in the highest resolution shell. In serially collected data, $I/\sigma(I)$ is understood to be underestimated, and CC*=0.5 is suggested as a better resolution cut-off criterion.*

Supplementary Table 2: Structure solution and refinement

Resolution range (Å)	48.0-2.8
Completeness (%)	100
No. of reflections, working set	6223
No. of reflections, test set	655
Final R_{cryst}	18.6
Final R_{free}	24.9
No. of non-H atoms	
Protein	2100
Ion	0
Ligand	0
Water	2
Total	2102
R.m.s. deviations	
Bonds (Å)	0.006
Angles (°)	1.46
Average *B* factors (Å^2)	
Protein	97.25
Water	72.6
Ramachandran plot	
Most favored (%)	92.2
Allowed (%)	6.0

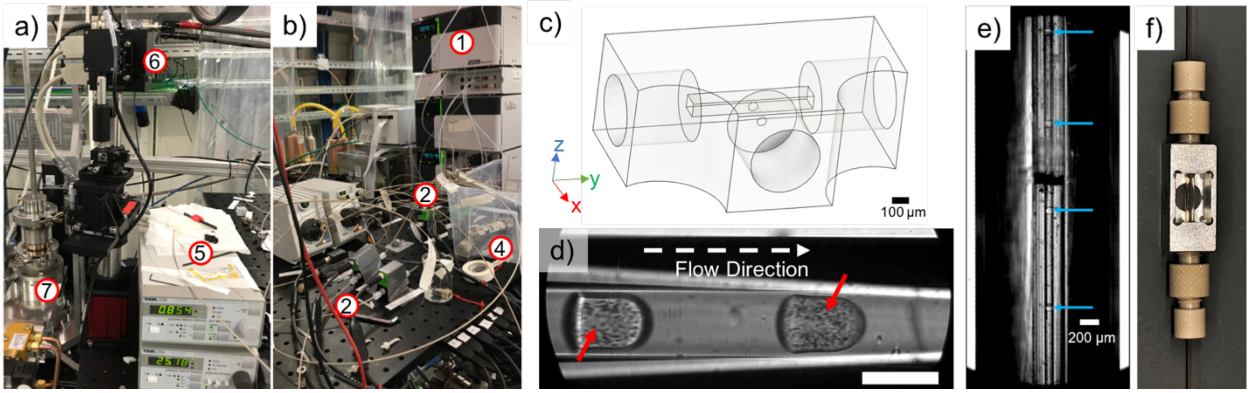

Supplementary Figure 1**: **Additional experimental illustrations. *a) and b) photographs of the set-up used for segmented droplet generation on top of the SPB/SFX chamber. Numbers indicate the components which are: 1. HPLC pumps, 2. flow rate sensors, 3. crystal suspension reservoir in an anti-settling device (not shown in the figure), 4. oil reservoir, 5. droplet generator, 6. droplet detector, 7. GDVN. c) CAD rendering of the droplet generator. d) Representative image of Photosystem I (PSI) crystals within droplets of mother liquor segmented by the oil phase. Droplets were generated at* Q_{aq} *= 4.5 µL/min and* Q_{oil} *= 12 µL/min and imaged in the capillary after the droplet generator. Dark dots in droplets, emphasized by red arrows, indicate crystals. For demonstration purposes, PSI crystals were used as they provide a high contrast for imaging. PSI crystals were prepared and grown as described previously.[1] Scale bar is 100 µm in d). e) and f) show the PicoClear union employed to jin the outlet capillary of the droplet generator with the liquid capillary transporting droplets to the nozzle tip. e) shows an image before and after the dead volume free union demonstrating droplet transfer from capillary to capillary. f) Assembled PicoClear union.*

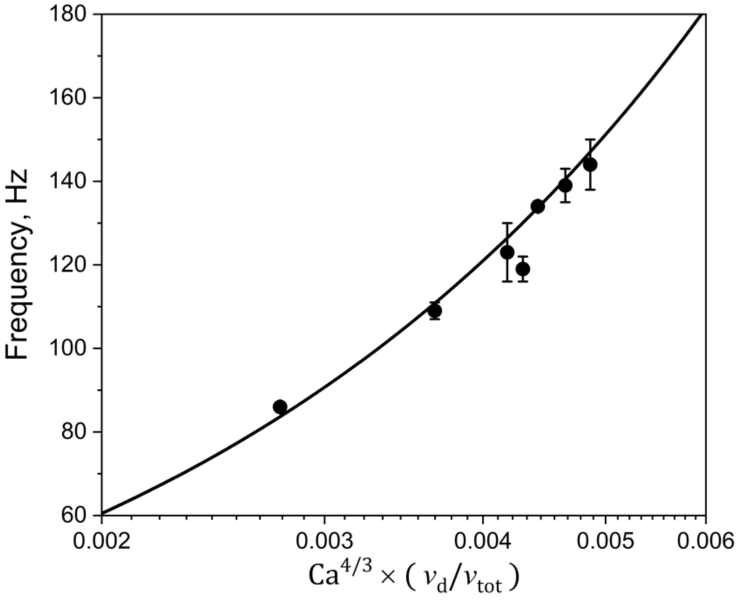

***Supplementary Figure 2**: **Segmented flow in a higher frequency range.** The droplet generator can also achieve frequencies between 80 and 150 Hz in agreement with equation 1 (main manuscript). This is an example for aqueous droplets of photosystem I mother liquor droplets generated in the perfluoinated oil. The factor K is 0.23 ± 0.01 m/s in this case and the error bars represent the standard deviation.*

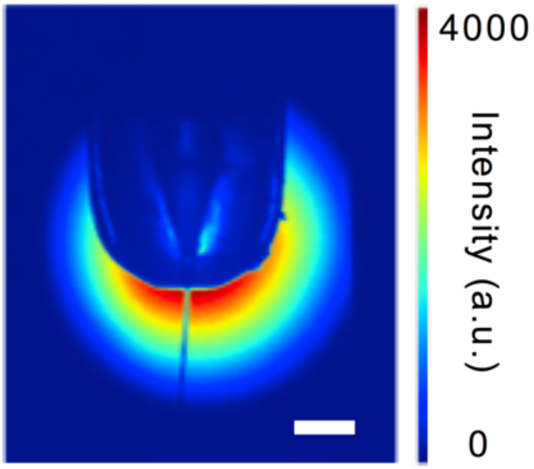

Supplementary Figure 3*: *Liquid jet in the SPB/SFX chamber. *False color image of a jet from a surface treated GDVN in the SPB/SFX vacuum chamber during oil injection. Scale bar is 300 µm. The laser illumination is in-line with the camera as seen by the high intensity rings.*

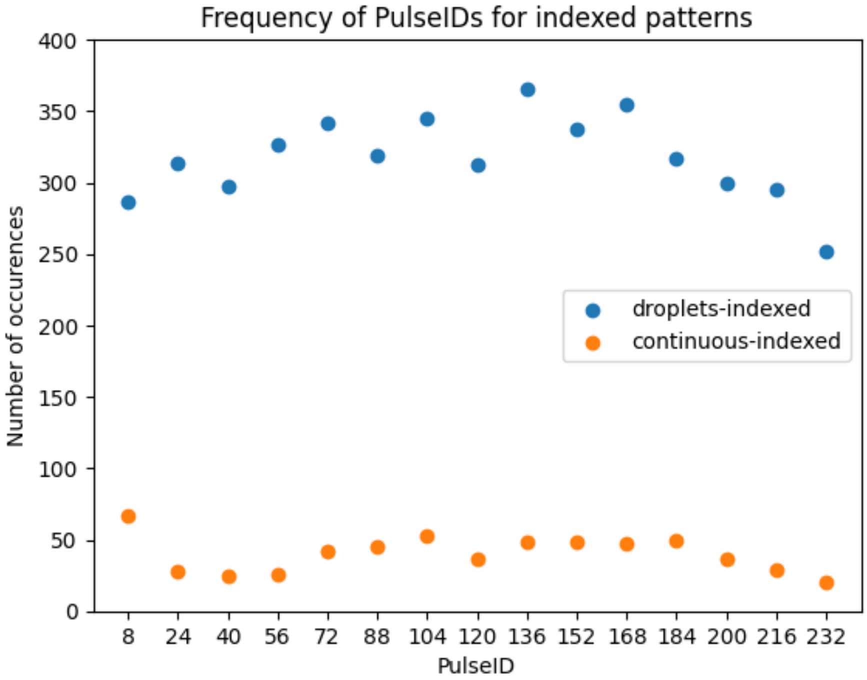

Supplementary Figure 4: Frequency of Pulse IDs for indexed patterns. *The frequency of unique pulse ID occurences is plotted for all indexed patterns obtained using droplet injection and continuous injection. There is no dependency of indexing on pulse ID, demonstrating that the jet speeds used in this experiment were fast enough for the 1.1 MHz repetition rate within the pulse trains.*

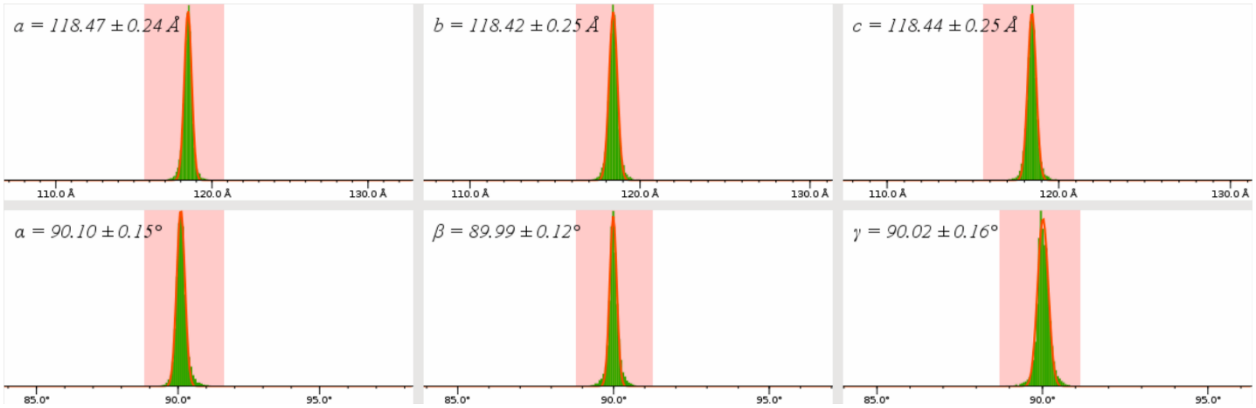

Supplementary Figure 5: Unit cell size distribution. *It is shown for the 15,777 indexed and integrated diffraction patterns. A Gaussian function was fit to each distribution and the mean calculated using cell_explorer from the CrystFEL software package.*

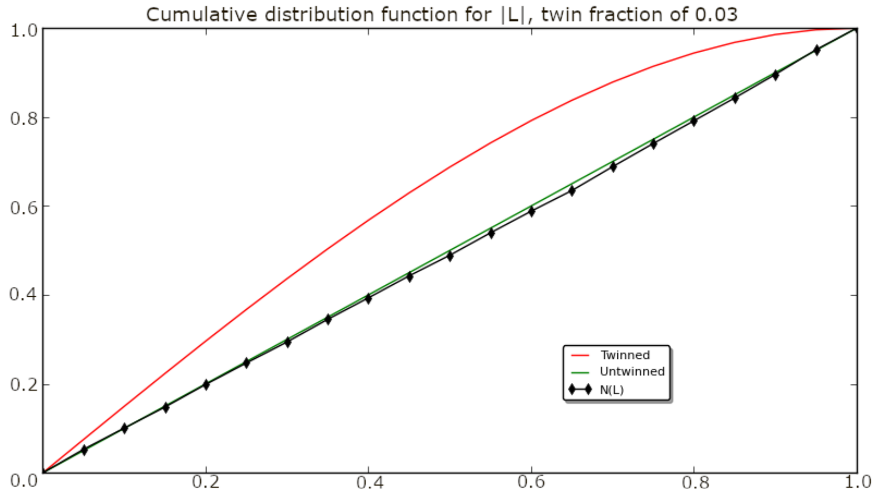

Supplementary Figure 6: L-test. *Results of this test show that the data is de-twinned and the indexing ambiguitiy associated with the cubic I23 spacegroup has been resolved.*

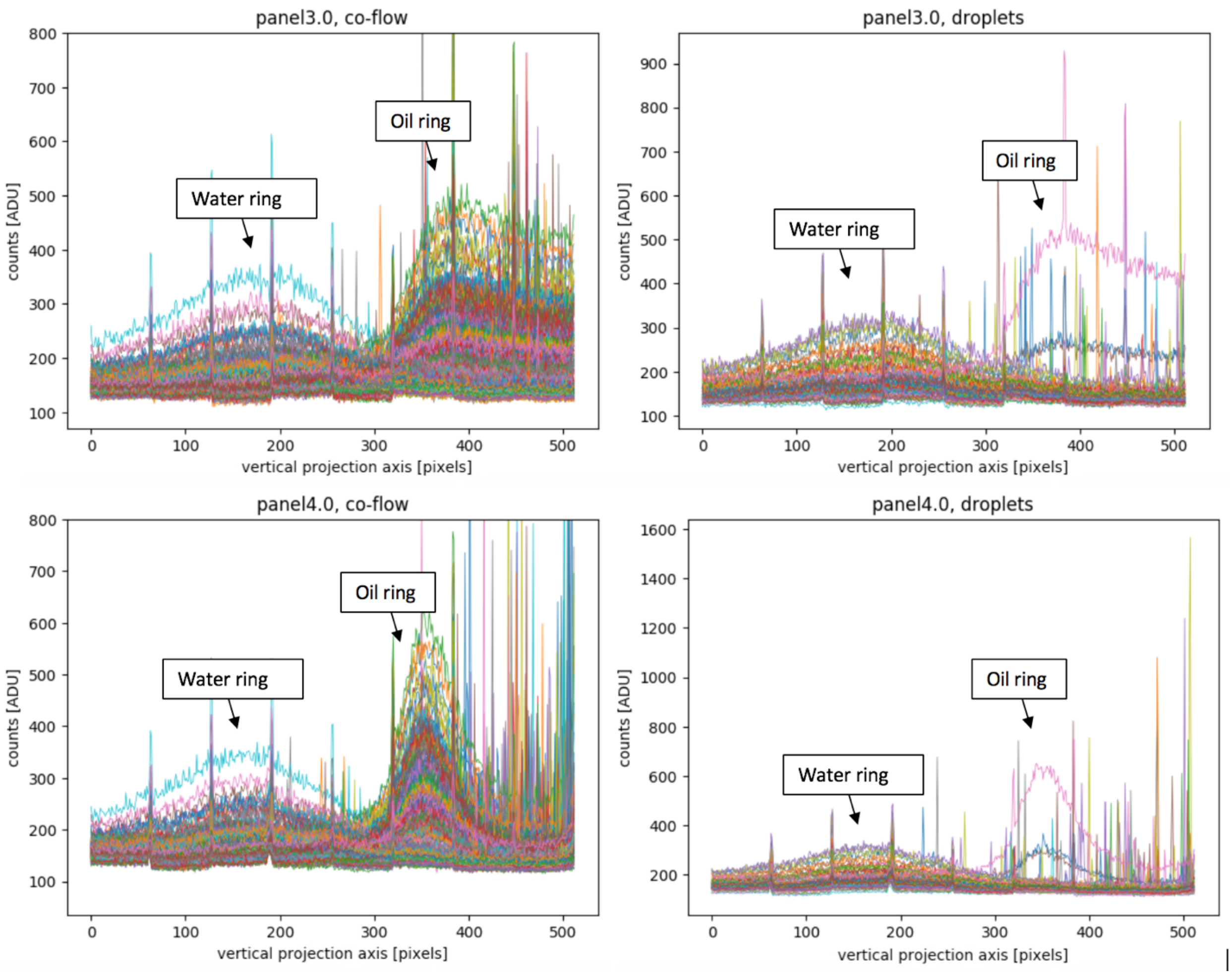

Supplementary Figure 7: Selected vertical projection of detector panels. *The vertical projection of the AGIPD panels 03 and 04 for a single run using a) droplet injection and b) co-flow of oil and aqueous crystal suspension. Diffuse scattering resulting for water and oil is indicated in each graph, clearly indicating that there is no major contribution from the oil scattering during the droplet injection. The rare occurances of the oil in the droplet injection case are attributed to instances where the droplet is hit at the edge where both oil and water are apparent.*

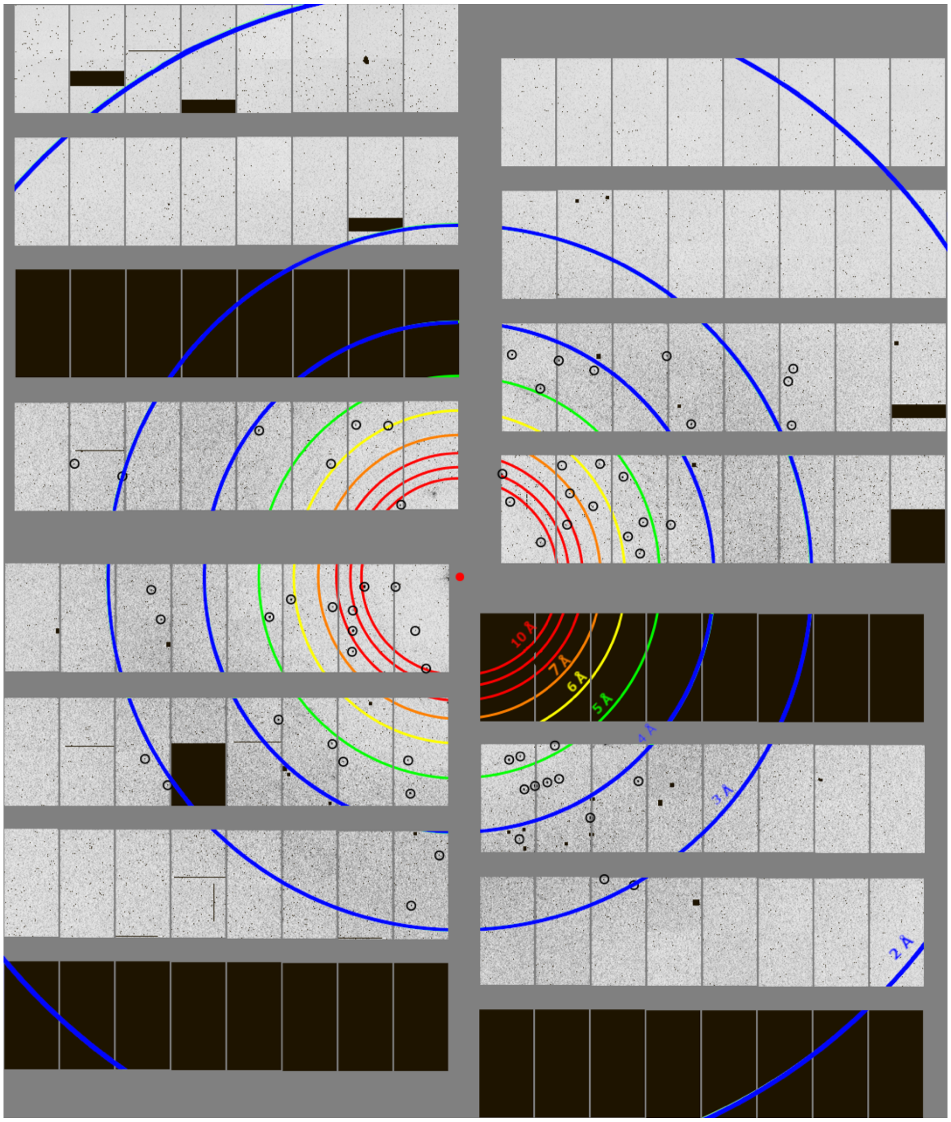

***Supplementary Figure 8**: **Representative diffraction pattern of a single KDO8PS microcrystal with resolution up to 2.80 Å.** Found peaks are highlighted with black circles as determined by Cheetah. Larger black regions and spots are masked out areas on the detector.*

***Supplementary Figure 9**: **KDO8PS in the pET-23d plasmid:** The vector map.*

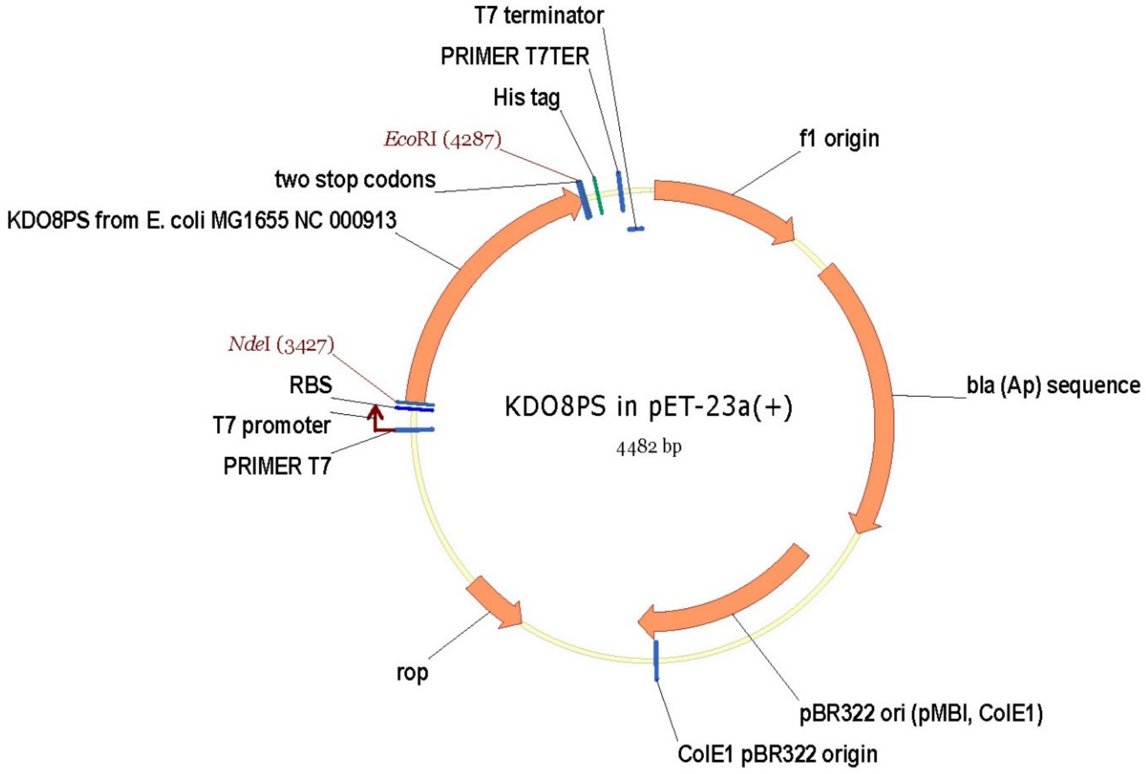

***Supplementary Figure 10**: **KDO8PS in the pET-23d plasmid:** The complete DNA sequence of the plasmid.*

```
LOCUS       KDO8PS\in\pET-23a(+)\DTH        4482 bp    DNA     circular      8-JUL-2020
DEFINITION
SOURCE
  ORGANISM
FEATURES             Location/Qualifiers
     rep_origin      12..467
                     /vntifkey="33"
                     /label=f1\origin
     CDS             599..1456
                     /vntifkey="4"
                     /label=bla\(Ap)\sequence
     rep_origin      2217..2217
                     /vntifkey="33"
                     /label=ColE1\pBR322\origin
     terminator      4411..4457
                     /vntifkey="43"
                     /label=T7\terminator
     protein_bind    4326..4343
                     /vntifkey="31"
                     /label=His\tag
     promoter        3348..3364
                     /vntifkey="30"
                     /label=T7\promoter
     CDS             3428..4279
                     /vntifkey="4"
                     /label=KDO8PS\from\E.\coli\MG1655\NC_000913
     misc_feature    4280..4285
                     /vntifkey="21"
                     /label=two\stop\codons
     RBS             3411..3420
                     /vntifkey="32"
                     /label=RBS
     CDS             complement(2648..2839)
                     /vntifkey="4"
                     /label=rop
     rep_origin      1604..2277
                     /vntifkey="33"
                     /label=pBR322\ori\(pMBI,\ColE1)
     primer          3348..3367
                     /vntifkey="27"
                     /label=PRIMER\T7
     primer          complement(4396..4414)
                     /vntifkey="27"
                     /label=PRIMER\T7TER
BASE COUNT     1086 a      1116 c      1163 g      1117 t
ORIGIN
        1 tggcgaatgg gacgcgccct gtagcggcgc attaagcgcg gcgggtgtgg tggttacgcg
       61 cagcgtgacc gctacacttg ccagcgccct agcgcccgct cctttcgctt tcttcccttc
      121 ctttctcgcc acgttcgccg gctttccccg tcaagctcta aatcgggggc tccctttagg
      181 gttccgattt agtgctttac ggcacctcga ccccaaaaaa cttgattagg gtgatggttc
      241 acgtagtggg ccatcgccct gatagacggt ttttcgccct ttgacgttgg agtccacgtt
      301 ctttaatagt ggactcttgt tccaaactgg aacaacactc aaccctatct cggtctattc
      361 ttttgattta taagggattt tgccgatttc ggcctattgg ttaaaaaatg agctgattta
      421 acaaaaattt aacgcgaatt ttaacaaaat attaacgttt acaatttcag gtggcacttt
      481 tcggggaaat gtgcgcggaa cccctatttg tttatttttc taaatacatt caaatatgta
      541 tccgctcatg agacaataac cctgataaat gcttcaataa tattgaaaaa ggaagagtat
      601 gagtattcaa catttccgtg tcgcccttat tccctttttt gcggcatttt gccttcctgt
      661 ttttgctcac ccagaaacgc tggtgaaagt aaaagatgct gaagatcagt tgggtgcacg
      721 agtgggttac atcgaactgg atctcaacag cggtaagatc cttgagagtt ttcgccccga
      781 agaacgtttt ccaatgatga gcacttttaa agttctgcta tgtggcgcgg tattatcccg
      841 tattgacgcc gggcaagagc aactcggtcg ccgcatacac tattctcaga atgacttggt
      901 tgagtactca ccagtcacag aaaagcatct tacggatggc atgacagtaa gagaattatg
      961 cagtgctgcc ataaccatga gtgataacac tgcggccaac ttacttctga caacgatcgg
     1021 aggaccgaag gagctaaccg cttttttgca caacatgggg gatcatgtaa ctcgccttga
     1081 tcgttgggaa ccggagctga atgaagccat accaaacgac gagcgtgaca ccacgatgcc
     1141 tgcagcaatg gcaacaacgt tgcgcaaact attaactggc gaactactta ctctagcttc
     1201 ccggcaacaa ttaatagact ggatggaggc ggataaagtt gcaggaccac ttctgcgctc
     1261 ggcccttccg gctggctggt ttattgctga taaatctgga gccggtgagc gtgggtctcg
     1321 cggtatcatt gcagcactgg ggccagatgg taagccctcc cgtatcgtag ttatctacac
     1381 gacggggagt caggcaacta tggatgaacg aaatagacag atcgctgaga taggtgcctc
```

```
1441 actgattaag cattggtaac tgtcagacca agtttactca tatatacttt agattgattt
1501 aaaacttcat ttttaattta aaaggatcta ggtgaagatc ctttttgata atctcatgac
1561 caaaatccct taacgtgagt tttcgttcca ctgagcgtca gaccccgtag aaaagatcaa
1621 aggatcttct tgagatcctt tttttctgcg cgtaatctgc tgcttgcaaa caaaaaaacc
1681 accgctacca gcggtggttt gtttgccgga tcaagagcta ccaactcttt ttccgaaggt
1741 aactggcttc agcagagcgc agataccaaa tactgtcctt ctagtgtagc cgtagttagg
1801 ccaccacttc aagaactctg tagcaccgcc tacatacctc gctctgctaa tcctgttacc
1861 agtggctgct gccagtggcg ataagtcgtg tcttaccggg ttggactcaa gacgatagtt
1921 accggataag gcgcagcggt cgggctgaac ggggggttcg tgcacacagc ccagcttgga
1981 gcgaacgacc tacaccgaac tgagatacct acagcgtgag ctatgagaaa gcgccacgct
2041 tcccgaaggg agaaaggcgg acaggtatcc ggtaagcggc agggtcggaa caggagagcg
2101 cacgagggag cttccagggg gaaacgcctg gtatctttat agtcctgtcg ggtttcgcca
2161 cctctgactt gagcgtcgat ttttgtgatg ctcgtcaggg gggcggagcc tatggaaaaa
2221 cgccagcaac gcggcctttt tacggttcct ggccttttgc tggccttttg ctcacatgtt
2281 ctttcctgcg ttatcccctg attctgtgga taaccgtatt accgcctttg agtgagctga
2341 taccgctcgc cgcagccgaa cgaccgagcg cagcgagtca gtgagcgagg aagcggaaga
2401 gcgcctgatg cggtattttc tccttacgca tctgtgcggt atttcacacc gcatatatgg
2461 tgcactctca gtacaatctg ctctgatgcc gcatagttaa gccagtatac actccgctat
2521 cgctacgtga ctgggtcatg gctgcgcccc gacacccgcc aacacccgct gacgcgccct
2581 gacgggcttg tctgctcccg gcatccgctt acagacaagc tgtgaccgtc tccgggagct
2641 gcatgtgtca gaggttttca ccgtcatcac cgaaacgcgc gaggcagctg cggtaaagct
2701 catcagcgtg gtcgtgaagc gattcacaga tgtctgcctg ttcatccgcg tccagctcgt
2761 tgagtttctc cagaagcgtt aatgtctggc ttctgataaa gcgggccatg ttaagggcgg
2821 ttttttcctg tttggtcact gatgcctccg tgtaaggggg atttctgttc atgggggtaa
2881 tgataccgat gaaacgagag aggatgctca cgatacgggt tactgatgat gaacatgccc
2941 ggttactgga acgttgtgag ggtaaacaac tggcggtatg gatgcggcgg gaccagagaa
3001 aaatcactca gggtcaatgc cagcgcttcg ttaatacaga tgtaggtgtt ccacagggta
3061 gccagcagca tcctgcgatg cagatccgga acataatggt gcagggcgct gacttccgcg
3121 tttccagact ttacgaaaca cggaaaccga agaccattca tgttgttgct caggtcgcag
3181 acgttttgca gcagcagtcg cttcacgttc gctcgcgtat cggtgattca ttctgctaac
3241 cagtaaggca accccgccag cctagccggg tcctcaacga caggagcacg atcatgcgca
3301 cccgtggcca ggacccaacg ctgcccgaga tctcgatccc gcgaaattaa tacgactcac
3361 tatagggaga ccacaacggt ttccctctag aaataatttt gtttaacttt aagaaggaga
3421 tatacatatg aaacaaaaag tggttagcat tggcgacatc aacgtagcaa atgacctgcc
3481 gttcgtactg tttggcggta tgaacgtgtt ggaatctcgc gatctggcga tgcgcatttg
3541 cgagcactac gtaactgtga cccagaaact gggtatccct tacgtgttca aagcctcttt
3601 tgacaaagcc aaccgctcct ccatccactc ttatcgtgga ccgggcctgg aagaagggat
3661 gaaaatcttc caggagttga agcagacttt tggcgtgaaa attatcaccg acgttcacga
3721 accaagtcag gcacagcccg ttgctgatgt cgtggatgtg attcagttgc cggcgtttct
3781 tgctcgccag actgacctgg ttgaagccat ggcgaaaacc ggtgcggtaa ttaacgtcaa
3841 gaaaccacag tttgtcagcc cgggacagat gggtaatatc gttgataaat tcaaagaagg
3901 cggcaacgaa aaagtgattc tttgcgatcg cggtgctaac ttcggctatg acaacctggt
3961 tgtcgatatg ctgggcttca gcattatgaa gaaagtgtct ggtaactcgc cggtgatttt
4021 cgacgtgacc cacgcactgc aatgccgcga tccgtttggc gcagcttccg gtggtcgtcg
4081 tgctcaggtg gctgagctgg cacgagccgg tatggcggta ggtctggcgg ggctgtttat
4141 tgaagcgcat ccggatccgg aacatgcgaa atgtgatggt ccatccgcgc tgccgctggc
4201 taaactggaa ccgttcctca agcagatgaa agcgattgat gatctggtga aggtttcga
4261 agaactggat accagcaagt aataagaatt cgagctccgt cgacaagctt gcggccgcac
4321 tcgagcacca ccaccaccac cactgagatc cggctgctaa caaagcccga aaggaagctg
4381 agttggctgc tgccaccgct gagcaataac tagcataacc ccttggggcc tctaaacggg
4441 tcttgagggg ttttttgctg aaaggaggaa ctatatccgg at
//
```

***Supplementary Figure 11**: **KDO8PS in the pET-23d plasmid:** The expressed protein sequence.*

```
LOCUS       Translation\of\KDO8PS\in\pET-23a(+)\DTH        284 aa                            8-JUL-2020
DEFINITION  Translation of a fragment of KDO8PS in pET-23a(+) DTH.
KEYWORDS    TRANSLATED.
SOURCE
  ORGANISM
FEATURES             Location/Qualifiers
     Region          1..284
                     /vntifkey="1000"
                     /label=KDO8PS\from\E.\coli\MG1655\NC_000913
ORIGIN
        1 mkqkvvsigd invandlpfv lfggmnvles rdlamriceh yvtvtqklgi pyvfkasfdk
       61 anrssihsyr gpgleegmki fqelkqtfgv kiitdvheps qaqpvadvvd viqlpaflar
      121 qtdlveamak tgavinvkkp qfvspgqmgn ivdkfkeggn ekvilcdrga nfgydnlvvd
      181 mlgfsimkkv sgnspvifdv thalqcrdpf gaasggrraq vaelaragma vglaglfiea
      241 hpdpehakcd gpsalplakl epflkqmkai ddlvkgfeel dtsk
//
```

Supplementary References:

1. Abdallah, B.G., Chao, T.C., Kupitz, C., Fromme, P. & Ros, A. Dielectrophoretic sorting of membrane protein nanocrystals. *ACS Nano* **7**, 9129-9137 (2013).
