# Supplementary material for: Segmented flow generator for serial crystallography at the European X-ray free electron laser
Source: Nat Commun. 2020 Sep 9;11:4511. doi: 10.1038/s41467-020-18156-7 (PMC7481229; doi:10.1038/s41467-020-18156-7)
Supplement: Supplementary file 2 — Reporting summary [file 41467_2020_18156_MOESM2_ESM.pdf]

nature research

Corresponding author(s): Alexandra Ros

Last updated by author(s): Jul 8, 2020

Reporting Summary

Nature Research wishes to improve the reproducibility of the work that we publish. This form provides structure for consistency and transparency in reporting. For further information on Nature Research policies, see Authors & Referees and the Editorial Policy Checklist .

Statistics

For all statistical analyses, confirm that the following items are present in the figure legend, table legend, main text, or Methods section.

n/a	Confirmed	
☐	☒	The exact sample size (*n*) for each experimental group/condition, given as a discrete number and unit of measurement
☐	☒	A statement on whether measurements were taken from distinct samples or whether the same sample was measured repeatedly
☒	☐	The statistical test(s) used AND whether they are one- or two-sided<br>*Only common tests should be described solely by name; describe more complex techniques in the Methods section.*
☒	☐	A description of all covariates tested
☒	☐	A description of any assumptions or corrections, such as tests of normality and adjustment for multiple comparisons
☐	☒	A full description of the statistical parameters including central tendency (e.g. means) or other basic estimates (e.g. regression coefficient) AND variation (e.g. standard deviation) or associated estimates of uncertainty (e.g. confidence intervals)
☒	☐	For null hypothesis testing, the test statistic (e.g. *F*, *t*, *r*) with confidence intervals, effect sizes, degrees of freedom and *P* value noted<br>*Give P values as exact values whenever suitable.*
☒	☐	For Bayesian analysis, information on the choice of priors and Markov chain Monte Carlo settings
☒	☐	For hierarchical and complex designs, identification of the appropriate level for tests and full reporting of outcomes
☒	☐	Estimates of effect sizes (e.g. Cohen's *d*, Pearson's *r*), indicating how they were calculated

Our web collection on statistics for biologists contains articles on many of the points above.

Software and code

Policy information about availability of computer code

Data collection	KDO8PS diffraction patterns were collected with the AGIPD detector through the EuXFEL's data collection system. Diffraction patterns were identified and calibrated using Cheetah and further processed with data analysis software as described in the manuscript.
Data analysis	Data was analyzed as described in the methods section of the manuscript, using publicly available software with appropriate references.

For manuscripts utilizing custom algorithms or software that are central to the research but not yet described in published literature, software must be made available to editors/reviewers. We strongly encourage code deposition in a community repository (e.g. GitHub). See the Nature Research guidelines for submitting code & software for further information.

Data

Policy information about availability of data

All manuscripts must include a data availability statement. This statement should provide the following information, where applicable:

- Accession codes, unique identifiers, or web links for publicly available datasets
- A list of figures that have associated raw data
- A description of any restrictions on data availability

The protein model and the associated structure factors have been deposited in the Protein Data Bank with accession code 6U57. Furthermore, the diffraction patterns have been uploaded to the Coherent X-ray Imaging Data Bank (https://cxidb.org) along with the stream file outlining the indexing parameters for the individually indexed patterns. A source data file is available for Figures 2 and 3, as well as Supplementary Figure 2.

Field-specific reporting

Please select the one below that is the best fit for your research. If you are not sure, read the appropriate sections before making your selection.

Life sciences study design

All studies must disclose on these points even when the disclosure is negative.

Sample size	This manuscript describes the structure determination of the protein KDO8PS. We have selected data to compare diffraction quality with continuous liquid jet injection as the newly developed droplet injection method. Comparable data was selected for the same crystallization batch of KDO8PS, for the same number of runs and thus same amount of raw data recorded for the two cases. The structure of KDO8PS was determined based on all available diffraction data collected during one beam time at the EUXFEL as detailed in the manuscript.
Data exclusions	n/a
Replication	n/a
Randomization	n/a
Blinding	n/a

Reporting for specific materials, systems and methods

We require information from authors about some types of materials, experimental systems and methods used in many studies. Here, indicate whether each material, system or method listed is relevant to your study. If you are not sure if a list item applies to your research, read the appropriate section before selecting a response.

Materials & experimental systems

n/a	Involved in the study	
☒	☐	Antibodies
☒	☐	Eukaryotic cell lines
☒	☐	Palaeontology
☒	☐	Animals and other organisms
☒	☐	Human research participants
☒	☐	Clinical data

Methods

n/a	Involved in the study	
☒	☐	ChIP-seq
☒	☐	Flow cytometry
☒	☐	MRI-based neuroimaging
